# Applying the RE-AIM implementation framework to evaluate fall prevention interventions in community dwelling adults with cognitive impairment: a review and secondary analysis

**DOI:** 10.1186/s12877-021-02376-7

**Published:** 2021-07-26

**Authors:** M. Racey, M. Markle-Reid, D. Fitzpatrick-Lewis, M. U. Ali, H. Gagné, S. Hunter, J. Ploeg, R. Sztramko, L. Harrison, R. Lewis, M. Jovkovic, D. Sherifali

**Affiliations:** 1 McMaster Evidence Review and Synthesis Team; and School of Nursing, Faculty of Health Sciences, McMaster University, Hamilton, Canada; 2 School of Nursing, Faculty of Health Sciences, McMaster University; and Scientific Director, Aging, Community and Health Research Unit, McMaster University, Hamilton, Canada; 3 McMaster Evidence Review and Synthesis Team; and Department of Clinical Epidemiology & Biostatistics, Faculty of Health Sciences, McMaster University, Hamilton, Canada; 4 Prevention, Ontario Neurotrauma Foundation, Toronto, Canada; 5 School of Physical Therapy, University of Western Ontario, London, Canada; 6 School of Nursing, Faculty of Health Sciences, McMaster University; and Aging, Community and Health Research Unit, McMaster University, Hamilton, Canada; 7 Geriatric Medicine, McMaster University, Hamilton, Canada; 8 Caregiver Partner, Hamilton, Canada; 9 McMaster Evidence Review and Synthesis Team, Hamilton, Canada; 10 Director, McMaster Evidence Review and Synthesis Team; and School of Nursing, Faculty of Health Sciences, McMaster University, Hamilton, Canada

**Keywords:** Fall prevention, Cognitive impairment, Older adults, RE-AIM, Implementation

## Abstract

**Background:**

Cognitive impairment (CI) is a risk factor for falls due to environmental or living settings, balance, gait and vision impairments, as well as medications. While previous systematic reviews have focused on the effectiveness of fall prevention programs in adults with cognitive impairment, very limited information is available on their implementation. This review examines what aspects of fall prevention interventions for community-dwelling adults with CI have been reported using the Reach, Effectiveness, Adoption, Implementation, and Maintenance (RE-AIM) framework to support successful implementation.

**Methods:**

We examined the included studies from our systematic review, which searched 7 databases for primary and secondary fall prevention interventions involving community-dwelling adults ≥50 years with mild to moderate CI. Reviewers screened citations and extracted data for study characteristics and the 5 dimensions (62 criteria) of the RE-AIM framework.

**Results:**

Twelve randomized or clinical controlled trials (RCTs/CCTs) consisting of 8 exercise interventions, 3 multifactorial interventions, and 1 medication treatment were included in the review. Only 4 of 62 criteria were reported by all 12 included studies and 29 criteria were not reported by any of the studies. Five of the included studies reported on 20 or more of the 62 possible RE-AIM criteria and 3 of these studies self-identified as “feasibility” studies. While Reach was the best-reported construct by the included studies, followed by Effectiveness and Implementation, the criteria within the Adoption and Maintenance constructs were rarely mentioned by these studies. In general, there was also wide variation in how each of the criteria were reported on by study authors.

**Conclusion:**

Based on the reporting of RE-AIM components in this review, we are unable to make connections to successful intervention components and thus practice-based recommendations for fall prevention in those with CI. The lack of detail regarding implementation approaches greatly limits the interpretation and comparisons across studies to fully inform future research efforts.

**Supplementary Information:**

The online version contains supplementary material available at 10.1186/s12877-021-02376-7.

## Background

Falls result in adverse outcomes for both individuals and their caregivers and families due to injuries, health complications, and decreased quality of life [1, 2]. Falls also cost our public health system as they are the leading cause of injury-related admissions to acute care hospitals and in-hospital deaths. With an aging population the cost of fall injuries to seniors in Canada is estimated to rise from $2.4 billion a year in direct healthcare costs [3] to $240 billion by 2040 [4]. Similar impacts on the healthcare system are seen in other countries, such as the United States of America, where each year about $50 billion is spent on medical costs related to non-fatal fall injuries and $754 million is spent related to fatal falls [5].

Cognitive impairment is a well-recognized risk factor for falls due to multifactorial reasons, including the environment or living settings, balance, gait and vision impairments, as well as medications leading to an increased state of confusion [6–8]. Older adults with dementia fall two to three times more than cognitively healthy older adults [9] and 60–80% of people with dementia fall annually [6]. However, the risk factors for falls in adults with cognitive impairment are unique [10], limiting the ability of practitioners and clinicians to take what is known regarding fall prevention in cognitively healthy individuals [11–15] and translate these findings to patients with cognitive impairment [16]. The result is that little evidence is available to clinicians and health practitioners to guide practice to reduce falls in community-dwelling adults with cognitive impairment, specifically.

While it has been recognized that fall prevention interventions need to address multifactorial intrinsic and extrinsic risk factors and the body of evidence is growing in this population [6], the applicability and implementation of such fall prevention interventions to older adults with cognitive impairment is undetermined. Previous systematic reviews have focused on the effectiveness of fall prevention programs in adults with cognitive impairment [6, 17–19], but very limited information is available on their implementation. This gap in the research for reducing falls in those with cognitive impairment and the need for more detailed reporting of these interventions has been recognized previously [6] and still goes unmet. Knowledge of the factors that influence the implementation of fall prevention interventions may enhance their adoption and use [16].

The Reach, Effectiveness, Adoption, Implementation, and Maintenance (RE-AIM) framework was developed to improve the reporting of essential program elements that can advance the sustainable adoption and implementation of effective, generalizable, evidence-based interventions [20]. While the RE-AIM framework has been used in the planning, translation, dissemination, and evaluation of individual fall prevention interventions for older adults [21–23], to date, no fall prevention systematic review has summarized the proportion of studies which report on various RE-AIM dimensions or components. Many implementation theories exist, however, the advantage of using the RE-AIM framework is that it provides an outline for relevant intervention outcomes, such as the effectiveness of the intervention and their implementation strategies, but also on the potential for further application, adoption, and scaling of these interventions for long-term maintenance of successful programs. This, in turn, will help individuals to select effective interventions with the potential for broader impact.

This paper builds on a related systematic review in which we investigated the effectiveness of fall prevention interventions in community-dwelling adults with mild to moderate cognitive impairment [24]. In this paper, we examine the application and reporting of various dimensions of the RE-AIM framework in the studies included in the systematic review to inform future practice-based implementation research of such fall prevention initiatives.

## Methods

This review is a secondary research question to a systematic review and meta-analysis [24]. This paper focused on the second research question from a registered protocol (PROSPERO-CRD42020210916) and examines the included studies from our systematic review of the fall prevention literature in community-dwelling adults diagnosed with mild to moderate cognitive impairment [24].

### Search strategy

The search terms, databases, and strategy were developed in consultation with a research librarian at McMaster University and informed by previous systematic reviews [2, 19, 25] (Additional File 1). We searched MEDLINE, Embase, PsycINFO, Cochrane Central Register of Controlled Trials (CENTRAL), Cumulative Index of Nursing and Allied Health Literature (CINAHL), Web of Science, and Science Direct up to April 2020 and manually searched reference lists of relevant reviews and included studies for citations not captured in our search. Results from the search were deduplicated, and citations were uploaded to a secure internet-based platform for screening (DistillerSR, Evidence Partners Inc., Ottawa, Canada).

### Eligibility criteria

The eligibility criteria were established for the primary research question from the original review [24] and have been previously explained in detail. Briefly, we included fall prevention interventions in community-dwelling adults (aged 50+) with mild and/or moderate cognitive impairment (CI). CI had to be assessed by a valid and reliable tool, diagnosis, or medical report, and/or clearly identified and described by study authors. For our review, community dwelling included individuals living in a community setting (with or without caregiver support) and can include different locations/settings, however, we excluded those living in retirement homes, nursing homes, long-term care homes, acute care, or hospital settings where they may receive full-time support and care for activities of daily living. We also excluded studies of older adults with severe CI.

The main purpose of the intervention had to be either primary or secondary fall prevention as defined by the Institute for Work and Health [26]. Studies must have been available in English, peer-reviewed, and comprised of interventions with a control group (randomization was not required). For our review, a control group was defined as treatment as usual, usual care (i.e., no change to usual activities), or minimal contact (an intervention not thought to reduce falls such as general health education or social visits). We did not include or exclude studies based on outcomes measured.

### Data extraction

A team of researchers conducted the screening and data extraction (M.R., D.F.L., R.L., M.J.). A minimum of two reviewers were required to independently and in duplicate screen titles and abstracts of all potentially eligible studies. Articles marked for inclusion by either team member went on to full-text screening which was completed independently and in duplicate by 2 team members and required consensus for inclusion or exclusion. We developed, piloted, and deployed standardized forms for data extraction. Two team members independently completed full data extraction of study characteristics (setting, sample size, inclusion and exclusion criteria, characteristics of participants, type of intervention (categories based on existing literature [27] and taxonomies [28]), and experimental and control components) and the 5 dimensions (62 criteria) of RE-AIM [20, 29]. Studies were also assessed for Risk of Bias, which can be found in our complementary review [24].

For the RE-AIM data extraction, reviewers used an adapted extraction tool designed specifically for conducting systematic reviews using RE-AIM [29]. Reach was evaluated by 12 criteria including descriptions of the target population, inclusion, and exclusion criteria, who participated or was exposed to the intervention, participation rates, and characteristics of those who participated and those who did not. Effectiveness (or efficacy) was evaluated by 9 RE-AIM criteria including reporting of mediators and moderators, how data were treated, quality of life, unintended or negative consequences, and attrition. Adoption was assessed at both the setting and provider/staff levels by 10 and 11 criteria, respectively. The Adoption construct included criteria such as the number and proportion of setting and staff members who agreed to participate in delivering the intervention, description of target locations or providers, how these settings and staff members were recruited, and how representative they were of the intended audience in terms of setting and staff. Implementation was assessed by 11 criteria as our research team added 2 criteria (engagement to inform intervention development and tailoring of intervention). Other existing criteria included whether interventions were theory-based, detailed descriptions of intervention protocols and how well these protocols were adhered to (fidelity), costs, and the completion rates of intervention participants. Maintenance was evaluated by 9 RE-AIM criteria including sustained impact of the intervention after termination for the participants and at the setting/staff level.

We included two additional components from the template for intervention description and replication (TIDieR) checklist and guide [30], as these are not covered by RE-AIM: details about tailoring the intervention for participants and the engagement of practitioners, participants, and/or caregivers in the development of the intervention. We also considered patient experience, caregiver outcomes and provider experience in extraction as additional data of interest. See Additional File 2 for RE-AIM components and definitions of each criteria. The lead researcher of this review (M.R.) resolved conflicts.

## Results

**Fig. 1:**
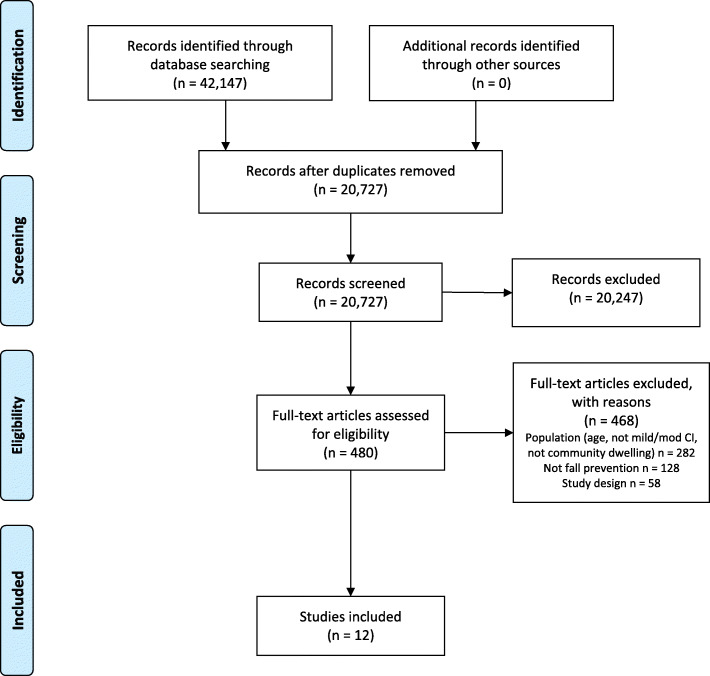
PRISMA Flowchart

From 20,727 citations, we assessed 480 full-text articles for eligibility, and included 10 randomized controlled trials (RCTs) and 2 clinical controlled trials (CCTs) in this review (Fig. 1). A full list of excluded studies with reasons is available upon request. The majority of studies (*n* = 8) were exercise interventions [31–38], while 3 were multifactorial [1, 39, 40], and 1 provided medication treatment [41]. Five of the 12 included studies were self-described as “feasibility” studies [1, 31, 32, 36, 37]. Overall, the studies were published from 2010 to 2020. Characteristics of the included studies can be found in Table 1 and further demographic data from studies can be found in Additional File 3. A total sample of 509 community-dwelling adults with mild to moderate cognitive impairment were included in this review with a mean age ranging from 67.5 to 84.0 years and percentage of women in the studies ranging from 20 to 74%. All included studies had fewer than 122 participants with most studies consisting of less than 50 participants total (*n* = 8). Studies were conducted across the globe in North America, South America, Europe, Asia, and Australia, and intervention duration was between 4 weeks to 1 year, but most studies (*n* = 8) were between 12 weeks and 6 months in duration. While all participants were community-dwelling, interventions took place in a combination of settings including home (*n* = 6), community centres (*n* = 3), and research centres (*n* = 2).

**Table 1:** Fall Prevention Study Details

Study, Year (reference)	N^**1**^	Age, mean y (SD)	Gender^**2**^ (F/M, %)	Cognitive Impairment Tool & Baseline Score	Study Design	Intervention Duration^**3**^	Intervention Category & Setting	Control	Harms
Varriano, 2020 [31]	7	O: 79.1 (6.7)	57/43	MoCA O: 21.2 (2.9)	RCT*	12 weeks	Exercise; vestibular exercises N/R	Usual care	Falls, but unclear if due to intervention
Goldberg, 2019 [32]	60	O: 76 (range 65–91)	43/57	MMSE O: 25.6 (3.1); I: 24.8 (3.6); 26.2 (3.2); C: 25.9 (2.4)	RCT*	12 months	Exercise; Balance, strength, dual-task training, gait re-education Home-based	Single falls prevention assessment	19 recorded adverse events (5 non-serious but intervention related)
Padala, 2017 [33]	30	O: 73.0 (6.2); I: 72.1 (5.3); C: 73.9 (7.1)	37/63	MMSE O: 22.9 (2.2); I: 23.3 (2.2); C: 22.7 (2.3)	RCT	8 weeks	Exercise; Wii-fit (yoga, strength, aerobics, balance) Home-based	Self-paced walking program	None study related
Zieschang, 2017 [34]	122	I: 82.1 (6.6); C: 82.2 (6.7)	74/26^2^	MMSE I: 21.6 (2.9); C: 21.9 (3.3)	RCT	3 months	Exercise; progressive resistance and functional training (activities of daily living, balance, walking, gait) N/R	Seated motor training exercises	N/R
Sungkarat, 2017 [35]	66	I: 68.3 (6.7); C: 67.5 (7.3)	50/50	MoCA, MMSE I: MoCA: 21.2 (3.4), MMSE: 26.5 (1.7); C: MoCA: 20.4 (3.8), MMSE: 25.8 (2.3)	RCT	15 weeks	Exercise; Tai Chi Community-centre and home-based	Educational material covering information related to cognitive impairment and fall prevention	No adverse events found
Schwenk, 2016 [36]	22	O: 78.2 (8.7); I: 77.8 (6.9); C: 79.0 (10.4)	55/45	MoCA O: 23.3 (2.6); I: 23.3 (3.1); C: 22.4 (3.0)	RCT*	4 weeks	Exercise; Balance (ankle point-to-point reaching tasks and virtual obstacle-crossing tasks) Research centre	Usual care	No training-related adverse events occurred
Montero-Odasso, 2019 [41]	60	O: 75.28 (7.18); I: 73.45 (5.74); C: 77.24 (8.11)	45/55	MMSE, MoCA O: sMMSE: 27.47 (1.96), MoCA: 23.60 (2.52); I: sMMSE: 27.42 (2.19), MoCA: 23.19 (2.55); C: sMMSE: 27.52 (1.72), MoCA: 22.97 (2.37)	RCT	6 months	Medication or vitamin supplement; Donepezil Home-based	Placebo	No major adverse events requiring treatment were reported
Chen, 2018 [39]	30	I: 77.3 (9.4); C: 77.3 (10.0)	50/50	MMSE, CDR I: MMSE: 16.4 (7.3), CDR: 0.5 = 6, 1.0 = 6, 2.0 = 3; C: MMSE: 17.9 (3.7), CDR: 0.5 = 3, 1.0 = 9, 2.0 = 1	RCT	2 months	Multifactorial; Musical dual-task training (physical and cognitive tasks) Community/research centre	Non-musical cognitive tasks and walking exercises	No adverse events reported
Kim, 2017 [40]	30	I: 82.0 (4.6); C: 80.9 (3.4)	20/80	MMSE-Korea I: 15.5 (2.9); C: 15.6 (2.4)	CCT	12 weeks	Multifactorial; physical activities, cognitive activities, activities of daily living, music activities Community centre	Usual care	N/R
Wesson, 2013 [1]	22	I: 78.7 (4.2); C: 80.9 (5.0)	41/59	ACE-R, MMSE I: ACE-R: 67.8 (12.6), MMSE: 24.5 (3.1); C: ACE-R: 62.5 (14.2), MMSE: 22.5 (4.3)	RCT*	12 weeks	Multifactorial; strength and balance exercises, home hazard reduction Home-based	Usual care, health promotion brochures on fall prevention and home safety	No serious adverse events related to the intervention were reported. Minor complaints relating to stiffness, dizziness and mild joint pain (*n* = 4; 36%) were reported.
Suttanon, 2013 [37]	40	O: 81.90 (5.72); I: 83.42 (5.10); C: 80.52 (6.01)	63/37	MMSE I: 20.89 (4.74); C: 21.67 (4.43)	RCT*	6 months	Exercise; balance and strength exercises, walking program Home-based	Education and information sessions on the topic of dementia and ageing	There were no falls or other serious adverse events associated with the intervention
Hernandez, 2010 [38]	20	O: 78.5 (6.8); I: 77.7 (7.6); C: 84.0 (6.1)	N/R	MMSE I: 16.4 (6.7); C: 14.2 (5.1)	CCT	6 months	Exercise; stretching, weight training, circuits, dance, recreational activities, relaxation N/R	Usual care	N/R

*O* overall population; *I* intervention; *C* control; *N/R* not reported; *RCT* randomized controlled trial; *CCT* clinical (non-randomized) controlled trial. *MoCA* Montreal cognitive assessment; *MMSE* Mini Mental State Exam; *ACE-R* Addenbrooke’s cognitive examination – revised; CDR = Clinical Dementia Rating scale; * = self-declared feasibility study

1. Number of participants randomized to intervention; 2. Values for gender are based on reported baseline which may not equal N randomized but rather the number of participants who completed the intervention; 3. Not including follow-up, if applicable

### Overall RE-AIM summary

A summary of the RE-AIM results by each element can be found in Table 2 (detailed extraction results are available in Additional File 4). Each study reported on at least one of the 62 RE-AIM criteria; only 4 criteria were reported by all 12 included studies and 29 criteria were not reported by any of the studies. The 4 criteria reported by all studies were participant characteristics, sample size, and intervention details, consistent with CONSORT guidelines [42]. Five of the included studies reported on 20 or more of the 62 possible RE-AIM criteria [1, 32, 33, 37, 39] and three of these studies were ones that self-identified as “feasibility” studies [1, 32, 37]. The study that reported the most criteria (23 out of 62) was a randomized trial to test the feasibility of study components and acceptability of a home hazard reduction and balance and strength exercise fall prevention program [1].

**Table 2:** RE-AIM Criteria Included in Each Study

		Study (reference)												
**RE-AIM Element**	**Criteria**	**Varriano, 2020 (32)**	**Goldberg, 2019** **(33)**	**Padala, 2017 (34)**	**Zieschang, 2017** **(35)**	**Sungkarat, 2017** **(36)**	**Schwenk, 2016 (37)**	**Montero-Odasso, 2019 (42)**	**Chen, 2018 (40)**	**Kim, 2017 (41)**	**Wesson, 2013** **(1)**	**Suttanon, 2013** **(38)**	**Hernandez, 2010** **(39)**	**Total**
Reach	Described target population	x	x	x	x	x	x	x	x	x	x	x	x	**12**
	Demographic, behavioral information about target population	x	x	x	x	x	x	x	x	x	x	x	x	**12**
	Method to identify the target population	x	x	x	x	x	x		x		x	x	x	**10**
	Recruitment strategies	x	x						x		x	x	x	**6**
	Inclusion/exclusion criteria for individuals	x	x	x	x	x	x	x	x	x	x	x		**11**
	Eligible, invited (exposed to recruitment) potential participants	x	x	x	x	x	x	x	x		x	x		**10**
	Sample size	x	x	x	x	x	x	x	x	x	x	x	x	**12**
	Individual participation rate (sample size/eligible invited potential participants)	x	x	x	x	x	x	x	x		x	x		**10**
	Comparisons between the target population and the study sample				x									**1**
	Statistical comparisons between the target population and the study sample				x									**1**
	Cost of recruitment													**0**
	Qualitative methods to measure reach													**0**
Effectiveness	Report of mediators				x									**1**
	Report of moderators			x	x	x	x	x	x			x		**7**
	Intent-to-treat			x	x	x		x	x		x	x		**7**
	Imputation procedures			x		x			x		x	x		**5**
	Quality-of-life measures	x	x	x						x		x		**5**
	Unintended consequences measures/results	x	x	x		x	x	x	x		x	x		**9**
	Percent attrition (at program completion)	x	x	x	x	x	x	x	x		x	x	x	**11**
	Cost-effectiveness													**0**
	Qualitative methods to measure efficacy/effectiveness						x							**1**
Adoption, setting	Eligible, invited potential settings													**0**
	Number of participating settings		x			x	x			x				**4**
	Setting participation rate													**0**
	Description of the targeted location													**0**
	Inclusion/exclusion criteria of the setting													**0**
	Description of intervention location		x	x		x	x	x	x	x	x	x		**9**
	Method to identify the setting													**0**
	Comparisons between the targeted and participating settings													**0**
	Statistical comparisons between the targeted and participating settings													**0**
	Average number of persons served per setting													**0**
Adoption, provider/staff	Eligible, invited potential providers (staff)													**0**
	Number of participating providers (staff)													**0**
	Provider (staff) participation rate													**0**
	Method to identify target providers													**0**
	Level of expertise of providers		x		x	x			x		x	x		**6**
	Inclusion/exclusion criteria for providers													**0**
	Comparisons between targeted and participating providers (staff)													**0**
	Statistical comparisons between targeted and participating providers (staff)													**0**
	Measures of cost adoption													**0**
	Dissemination beyond originally planned													**0**
	Qualitative methods to measure adoption													**0**
Implementation	Theory-based								x	x	x	x		**4**
	Engagement to inform intervention		x	x							x	x		**4**
	Number of intervention contacts	x	x	x	x	x	x	x	x	x	x	x	x	**12**
	Timing of intervention contacts	x	x	x	x	x	x	x	x	x	x		x	**11**
	Duration of intervention contacts		x		x	x	x		x	x	x		x	**8**
	Extent protocol delivered as intended (fidelity)		x								x			**2**
	Consistency of implementation across settings or providers								x		x			**2**
	Tailoring of intervention		x		x						x	x		**4**
	Participant attendance/completion rates		x	x		x	x				x	x		**6**
	Measure of intervention cost													**0**
	Qualitative methods to measure implementation	x					x				x	x		**4**
Maintenance	Follow-up outcome measures at some duration after intervention termination	x		x	x									**3**
	Attrition/loss to follow-up of individuals			x										**1**
	Qualitative methods to measure individual maintenance of the intervention													**0**
	Intervention alignment with the organization’s mission													**0**
	Maintenance of the program after completion of the study													**0**
	Modifications made to the original program													**0**
	Institutionalization of the program in the setting or system													**0**
	Attrition/loss to follow-up of settings													**0**
	Qualitative methods to measure organizational maintenance/ sustainability													**0**
**TOTAL FOR STUDY**		**15**	**21**	**20**	**18**	**19**	**19**	**13**	**20**	**11**	**23**	**22**	**10**	

Note: x indicates RE-AIM item reported for completeness

### RE-AIM criteria

#### Reach

Of all the RE-AIM constructs, this was the most thoroughly reported on by the included studies as 7 of the 12 criteria were described by 10 or more of the studies. All studies described the target population, provided demographic and behavioural information about the target population, and the sample size of the study. However, demographic, and behavioural information of the participants was not reported consistently across studies, with studies reporting different sample characteristics. For example, while almost all studies reported on gender and age of their participants, some did not include any information about ethnicity/race (*n* = 10), socioeconomic status (*n* = 4), or chronic diseases (*n* = 6) of their target population. Most of the studies (*n* ≥ 10) included information about how they identified the target population [1, 31–39], inclusion/exclusion criteria [1, 31–37, 39–41], the number of invited participants [1, 31–37, 39, 41], and the participation rate [1, 31–37, 39, 41]. Recruitment strategies (not location), such as referrals and newspaper ads, were reported by 6 of the included studies [1, 31, 32, 37–39]. Only one study [34] compared the target population to the study sample, and noted that those who did not participate were older and had a higher disease burden. No studies provided any information on the cost of recruitment or qualitatively measured their reach.

#### Effectiveness

Overall, almost all studies (*n* = 11) reported the attrition rate at study completion [1, 31–39, 41] and 9 studies explicitly reported on any unintended results or adverse events [1, 31–33, 35–37, 39, 41]. No study reported any serious adverse events related to the intervention protocol/program. These adverse events included falls, stiffness, dizziness, and mild joint pain, all of which were eased by modifying the program or with continued participation in the intervention. Many studies reported moderators (*n* = 7) [33–37, 39, 41] and followed intention-to-treat protocols (*n* = 7) [1, 33–35, 37, 39, 41], but fewer reported on imputation procedures for missing data (*n* = 5) [1, 33, 35, 37, 39], and quality of life outcomes (*n* = 5) [31–33, 37, 40]. No studies addressed cost-effectiveness and only 1 study reported qualitative measures of effectiveness [36] using a Likert scale questionnaire with participants.

#### Adoption

Overall, adoption was very poorly reported by all studies in our review. Nine of the included studies described the intervention location (e.g. home, community centres and research centres) [1, 32, 33, 35–37, 39–41] and 4 reported the number of settings where the intervention was delivered [32, 35, 36, 40]. Six studies provided details on the level of expertise required for providers/staff members to implement the intervention [1, 32, 34, 35, 37, 39]. No adoption criteria (e.g. participation rates of settings or providers, inclusion/exclusion criteria of settings or providers, or comparisons across different settings or providers) were reported by any studies.

#### Implementation

All studies (*n* = 12) provided information on the number of intervention contacts/visits and 11 studies were explicit in the timing of these intervention visits in regard to when they occurred over the duration of the study [1, 31–36, 38–41]. The number of visits and when they occurred varied greatly across included studies and was sometimes poorly described resulting in uncertainty. Eight studies [1, 32, 34–36, 38–40] provided the duration of intervention visits either within the methods section of the paper or in the results when reporting how long participants took to complete intervention components (i.e., how long they participated in the exercise session). However, no studies reported on how long the measurement or outcome assessments took. Overall, participant attendance or completion rates and qualitative measures of implementation were not well reported by studies. Additionally, 4 studies [1, 37, 39, 40] specified a theory or framework that informed the development of the intervention and while no studies stated involvement of participants or caregivers in designing the intervention, 4 studies did include caregivers in implementation of intervention components [1, 32, 33, 37]. Only 4 studies [1, 32, 34, 37] purposefully tailored their intervention methods and delivery to the individual participants in their study through individual activity/exercise plans that may have been adjusted for age, illness-related deficits, and/or tailored and specialized programming. Fidelity [1, 32] and consistency of implementation of the intervention [1, 39] was rarely reported on (*n* = 2 for each criteria) and no studies measured the cost of the intervention.

#### Maintenance

This construct was poorly reported by all studies in this review. Beyond immediate post intervention measurements, only 3 studies assessed the sustainability of the intervention effects following completion of the intervention [31, 33, 34]. Further, 1 of these 3 studies reported the loss of participants at this follow-up time period [33]. No studies assessed or reported on any of the other criteria such as maintenance of the program, modifications made to maintain the program, alignment of the intervention with the organization’s mission, or any qualitative methods to measure maintenance/sustainability.

## Discussion

This paper builds on a related systematic review which investigated the effectiveness of fall prevention interventions in community-dwelling adults with mild to moderate cognitive impairment. In this paper, we examined the application and reporting of various dimensions of the RE-AIM framework in the studies included in the systematic review to inform future practice-based implementation research of fall prevention initiatives. Overall, we found a general lack of reporting on most RE-AIM criteria. While Reach was the best-reported construct by the included studies, followed by Effectiveness and Implementation, the criteria within the Adoption and Maintenance constructs were rarely mentioned in these studies. In general, the RE-AIM criteria were not applied consistently, with some studies selecting to apply only particular criteria.

The RE-AIM framework was developed to improve the balanced reporting of internal and external validity of behavioural interventions [20]. It is thought that the transparent and consistent reporting of interventions may lead to a better understanding of their potential public health impact. However, across different disciplines, reviews that have investigated the use of RE-AIM and its reporting have concluded insufficient and inconsistent information on RE-AIM dimensions which leads to gaps related to who, under what conditions, and how these interventions are successful [29]. The need for more detailed reporting of fall prevention interventions for those with cognitive impairment, has been recognized previously [6]. Our review confirms this gap still exists as there was a lack of reporting on the RE-AIM criteria by studies. This included components such as eligibility criteria, descriptions of interventions, number of participants recruited and randomized, adverse events or harms, among others. Given the adherence to standard reporting guidelines such as CONSORT [42], as required by many journals, there may be the opportunity to expand these documents and figures to increase transparency in reporting external validity [43].

From the included studies in the systematic review, we found that the components reflecting external validity (i.e., Adoption/Diffusion and Maintenance) were the most challenging criteria for data extraction. Adoption is specifically segmented at two levels, setting and staff, while Maintenance has criteria that relate to both individual and organizational level. Only one intervention was delivered at multiple sites and it was not designed to test or measure implementation of these different settings based on these criteria [32]. Not knowing differences in settings and staff makes it difficult to determine what criteria might be needed for a site to successfully deliver the intervention, who in a real-world setting is best suited to deliver the intervention, or what settings might be appropriate for translation. Further, maintenance was reported on by almost no studies which leaves questions about the sustainability of these interventions, especially given the short duration of the studies. Fall prevention interventions for cognitively impaired adults also lack specific and detailed methods regarding implementation which limits the ability to replicate these programs with fidelity and sustainability. The lack of reporting on these external validity components also negatively impacts the ability of researchers and practitioners to translate research results into evidence-based policy and practice and to push the field forward. Without consistent and comprehensive reporting, it is difficult to assess whether the effectiveness of interventions is due to the intervention itself or its implementation components. This makes it challenging to inform the science of what and how interventions work and whom they work best with, to improve the development of future interventions.

The results of this review are not surprising given how little attention issues critical to translating research findings to public health often receive when compared with intervention effectiveness. The true impact of interventions is often constrained by barriers to their effective implementation [16]. Reviews typically focus on the effectiveness of interventions; however, without adequately exploring the interventions approach to implementation, there is limited ability to disseminate findings and scale those interventions that are effective [44]. While some of the included evidence in the review were feasibility or pilot studies (42%), they were also deficient in reporting on RE-AIM criteria, even though three of the five feasibility studies had the three highest number of reported criteria [1, 32, 37]. Traditionally, effectiveness science and implementation science have been considered separate entities. Ideally, systematic reviews would be able to report on effectiveness of interventions and relate these findings to implementation and external validity constructs, which would improve clinical practice and inform healthcare policies [16, 45]. This hybrid model of merging effectiveness and implementation trials has been evolving over the last several decades in response to the need for bridging the gap from efficacy (i.e. clinical trials) to effectiveness (i.e. clinical practice) [46, 47]. The effectiveness and implementation-focused trials help us to understand the effects of treatments on health outcomes under ‘usual care’ conditions [47]. A lack of the studies in this review fulfilling the RE-AIM criteria may be reflective of a few considerations. At the researcher level scientists are not conceptualizing trails with implementation of scale up in mind [48], the lag between evolving methods and being implemented into research, or specifically with this field of research in fall prevention for cognitively impaired adults, it may be the lack of clarity on effective interventions as many of the included studies were small in nature and appeared to be more pragmatic studies. While implementation science is on the rise and increasing in popularity [45], the current state of the science and evidence base regarding fall prevention in community dwelling adults with cognitive impairment has room for improvement.

### Limitations

Although our search was comprehensive, we did not explicitly search grey literature and we limited our studies to English-language citations. We did verify our included studies with those of other similar reviews and our results align with previous research in this area. It is also important to recognize that this review is a secondary research question to a systematic review and meta-analysis for which the search was originally intended. This search and inclusion/exclusion criteria for screening was not designed to select studies for implementation trials or RE-AIM fall prevention interventions. As we were not able to correlate the reporting of the RE-AIM constructs and criteria with the effectiveness of the interventions, our review is only able to comment on the state of science and research with respect to fall prevention interventions in community-dwelling adults with cognitive impairment.

### Implications for practice and research

The gap in the research for reducing falls in those with cognitive impairment and the need for more detailed reporting of these interventions continues to go unmet despite calls by previous research [6], health practitioners and clinicians themselves. Based on the reporting of RE-AIM components in this review, we are unable to make connections to successful intervention components and thus recommendations for practice. Fall prevention research for cognitively impaired adults is not meeting the needs of health practitioners and clinicians and there is little evidence to inform scale-up effective interventions in the community setting. The lack of information regarding external validity greatly limits interpretation and comparisons across studies that are required to fully understand impact and to inform future research efforts [49, 50]. Once effectiveness is known, more consistent reporting of fall prevention interventions is needed to more successfully translate results into practice.

## Conclusions

Because of the unique risk factors for falls in cognitively impaired adults, it is important that research focuses on effective fall prevention interventions for this high risk group. Researchers, health practitioners and clinicians have highlighted this gap and the need for research to inform evidence-based best practices and recommendations. However, there remains inadequate reporting of fall prevention interventions, especially regarding implementation guidance to health practitioners and clinicians for such interventions. These gaps in reporting of intervention components related to the RE-AIM framework and a lack of high quality studies limits the ability to translate research into practice. More standardized reporting on external validity is needed to determine whether fall prevention interventions or their components can be effectively delivered, in what setting, by whom it can be delivered, and whether it is sustainable in practice.

## Supplementary Information

**Additional file 1.**

**Additional file 2.**

**Additional file 3.**

**Additional file 4.**

## Data Availability

The main study data is the data extraction materials and quality ratings of included papers, most of which are included in the manuscript tables. Any other supporting data relating to this review is available from the authors.
