# Supplementary material for: Applying the RE-AIM implementation framework to evaluate fall prevention interventions in community dwelling adults with cognitive impairment: a review and secondary analysis
Source: BMC Geriatr. 2021 Jul 26;21:441. doi: 10.1186/s12877-021-02376-7 (PMC8314446; doi:10.1186/s12877-021-02376-7)
Supplement: Supplementary file 1 — Additional file 1. [file 12877_2021_2376_MOESM1_ESM.pdf]

Additional File 1
COGNITIVE IMPAIRMENT AND FALLS STRATEGIES
April 24, 2020

MEDLINE

Database: OVID Medline Epub Ahead of Print, In-Process & Other Non-Indexed Citations, Ovid MEDLINE(R) Daily and Ovid MEDLINE(R) 1946 to Present
Search Strategy:
----
1 (falls or faller or fall-related injury or fall prevention or falling or fell or slip* or trip* or stumble* or tumble*).ti,ab,kw. (390522)
2 Accidental Falls/ (23785)
3 exp Hip Fractures/ (23653)
4 hip fracture?.ti,ab,kw. (16089)
5 or/1-4 (425636)
6 exp cognition disorders/ or delirium/ or exp dementia/ or exp dyslexia, acquired/ (233743)
7 (cognitive* adj3 (impair* or dysfunction* or disorder?)).tw. (89832)
8 (dementia or alzheimer* or delirium or Lewy body or lewy bodies or acquired dyslexia).ti,ab,kw. (228229)
9 or/6-8 (357690)
10 5 and 9 (8716)
11 exp aged/ (3081904)
12 middle aged/ (4299639)
13 (adult? or individual? or elderly or middle aged or patient? or participant?).tw. (8674917)
14 or/11-13 (10317241)
15 10 and 13 (6319)

EMBASE

Search Strategy:
----
1 (falls or faller or fall-related injury or fall prevention or falling or fell or slip* or trip* or stumble* or tumble*).ti,ab,kw. (489853)
2 falling/ (39901)
3 exp hip injury/ (45150)
4 hip fracture?.ti,ab,kw. (23268)
5 or/1-4 (551697)
6 exp cognitive defect/ (482427)
7 exp delirium/ (30412)
8 exp alexia/ (1351)
9 (cognitive* adj3 (impair* or dysfunction* or disorder?)).tw. (140048)
10 (dementia or alzheimer* or delirium or Lewy body or lewy bodies or acquired dyslexia).ti,ab,kw. (327336)
11 or/6-10 (584438)
12 5 and 11 (17973)
13 middle aged/ (1489589)
14 aged/ or frail elderly/ or very elderly/ (2940516)
15 (adult? or individual? or elderly or middle aged or patient? or participant?).tw. (11970503)

16 or/13-15 (12948341)
17 12 and 16 (14171)

PSYCINFO
Database: APA PsycInfo <1806 to April Week 2 2020>
Search Strategy:
----
1 (falls or faller or fall-related injury or fall prevention or falling or fell or slip* or trip* or stumble* or tumble*).ti,ab. (46759)
2 falls/ (2827)
3 hips/ and injuries/ (341)
4 hip fracture?.ti,ab. (952)
5 or/1-4 (47762)
6 cognitive impairment/ (36298)
7 exp dementia/ (76142)
8 alzheimer's disease/ (46104)
9 delirium/ (3292)
10 (dementia or alzheimer* or delirium or Lewy body or lewy bodies or acquired dyslexia).ti,ab. (101697)
11 (cognitive* adj3 (impair* or dysfunction* or disorder?)).tw. (55353)
12 or/6-11 (148802)
13 5 and 12 (2825)

COCHRANE

ID	Search	Hits
#1	MeSH descriptor: [Accidental Falls] explode all trees	1413
#2	MeSH descriptor: [Hip Fractures] explode all trees	1581
#3	falls or faller or fall-related injury or fall prevention or falling or fell or slip* or trip* or stumble* or tumble*	40978
#4	hip fracture*	5784
#5	#1 or #2 or #3 or #4	46088
#6	MeSH descriptor: [Cognition Disorders] explode all trees	4913
#7	MeSH descriptor: [Dementia] explode all trees	5736
#8	MeSH descriptor: [Delirium] explode all trees	710
#9	MeSH descriptor: [Dyslexia, Acquired] explode all trees	10
#10	dementia or alzheimer* or delirium or Lewy body or lewy bodies or acquired dyslexia	27884
#11	(cognitive* near/3 (impair* or dysfunction* or disorder?))	14757
#12	#6 or $7 or #8 or #9 or #10 or #11	461299
#13	#5 and #12	20701
#14	MeSH descriptor: [Middle Aged] explode all trees	9402
#15	MeSH descriptor: [Aged] explode all trees	7469
#16	adult? or individual? or elderly or middle aged or patient? or participant?	1217342
#17	#14 or #15 or 16	217836
#18	#13 and #17	8654

WEB OF SCIENCE

Search History:

Set	Results	Save History / Create AlertOpen Saved History	Edit Sets	Combine Sets AND OR Combine	Delete Sets Select All Delete
# 3	7,582	#2 OR #1 *Indexes=SCI-EXPANDED, SSCI, A&HCI, CPCI-S, CPCI-SSH, BKCI-S, BKCI-SSH, ESCI, CCR-EXPANDED, IC Timespan=All years*	Edit		
# 2	839	TI=(cognitive impairment OR Alzheimer* OR dementia OR delirium or acquired dyslexia) AND TI= (fall* OR hip fracture*) *Indexes=SCI-EXPANDED, SSCI, A&HCI, CPCI-S, CPCI-SSH, BKCI-S, BKCI-SSH, ESCI, CCR-EXPANDED, IC Timespan=All years*	Edit		
# 1	7,582	TS=(cognitive impairment OR Alzheimer* OR dementia OR delirium or acquired dyslexia) AND TS= (fall* OR hip fracture*) *Indexes=SCI-EXPANDED, SSCI, A&HCI, CPCI-S, CPCI-SSH, BKCI-S, BKCI-SSH, ESCI, CCR-EXPANDED, IC Timespan=All years*	Edit		

SCIENCE DIRECT

Cognitive impairment and falls
Dementia and falls
Delirium and falls
Acquired dyslexia and falls
Alzheimer and falls

Friday, April 24, 2020 2:10:26 PM

#	Query	Limiters/Expanders	Last Run Via	Results
S15	S11 AND S14	Expanders - Apply equivalent subjects Search modes - Boolean/Phrase	Interface - EBSCOhost Research Databases Search Screen - Advanced Search Database - CINAHL	6,114
S14	S12 OR S13	Expanders - Apply equivalent subjects Search modes - Boolean/Phrase	Interface - EBSCOhost Research Databases Search Screen - Advanced Search Database - CINAHL	3,552,831
S13	TX (adult* or individual* or elderly or middle aged or patient* or participant*)	Expanders - Apply equivalent subjects Search modes - Boolean/Phrase	Interface - EBSCOhost Research Databases Search Screen - Advanced Search Database - CINAHL	3,552,740
S12	(MH "Aged+") OR (MH "Middle Age")	Expanders - Apply equivalent subjects Search modes - Boolean/Phrase	Interface - EBSCOhost Research Databases Search Screen - Advanced Search Database - CINAHL	1,340,547
S11	S5 AND S10	Expanders - Apply equivalent subjects	Interface - EBSCOhost	6,835

		Search modes - Boolean/Phrase	Research Databases Search Screen - Advanced Search Database - CINAHL	
S10	S6 OR S7 OR S8 OR S9	Expanders - Apply equivalent subjects Search modes - Boolean/Phrase	Interface - EBSCOhost Research Databases Search Screen - Advanced Search Database - CINAHL	148,266
S9	TX (cognitive* n3 (impair* or dysfunction* or disorder*))	Expanders - Apply equivalent subjects Search modes - Boolean/Phrase	Interface - EBSCOhost Research Databases Search Screen - Advanced Search Database - CINAHL	36,921
S8	TX (dementia or alzheimer* or delirium or Lewy body or lewy bodies or acquired dyslexia)	Expanders - Apply equivalent subjects Search modes - Boolean/Phrase	Interface - EBSCOhost Research Databases Search Screen - Advanced Search Database - CINAHL	111,590
S7	(MH "Dyslexia, Pure (Acquired)") OR (MH "Dyslexia, Surface (Acquired)")	Expanders - Apply equivalent subjects Search modes - Boolean/Phrase	Interface - EBSCOhost Research Databases Search Screen - Advanced Search Database - CINAHL	10
S6	(MH "Cognition Disorders+") OR (MH "Dementia+")	Expanders - Apply equivalent subjects Search modes -	Interface - EBSCOhost Research Databases	107,698

	OR (MH "Delirium")	Boolean/Phrase	Search Screen - Advanced Search Database - CINAHL	
S5	S1 OR S2 OR S3 OR S4	Expanders - Apply equivalent subjects Search modes - Boolean/Phrase	Interface - EBSCOhost Research Databases Search Screen - Advanced Search Database - CINAHL	146,614
S4	TX hip fracture*	Expanders - Apply equivalent subjects Search modes - Boolean/Phrase	Interface - EBSCOhost Research Databases Search Screen - Advanced Search Database - CINAHL	13,333
S3	TX falls or faller or fall-related injury or fall prevention or falling or fell or slip* or trip* or stumble* or tumble*	Expanders - Apply equivalent subjects Search modes - Boolean/Phrase	Interface - EBSCOhost Research Databases Search Screen - Advanced Search Database - CINAHL	135,201
S2	(MH "Hip Fractures+")	Expanders - Apply equivalent subjects Search modes - Boolean/Phrase	Interface - EBSCOhost Research Databases Search Screen - Advanced Search Database - CINAHL	10,625
S1	(MH "Accidental Falls")	Expanders - Apply equivalent subjects Search modes -	Interface - EBSCOhost Research Databases	24,279

	Boolean/Phrase	Search Screen - Advanced Search<br>Database - CINAHL
