# Supplementary material for: Applying the RE-AIM implementation framework to evaluate fall prevention interventions in community dwelling adults with cognitive impairment: a review and secondary analysis
Source: BMC Geriatr. 2021 Jul 26;21:441. doi: 10.1186/s12877-021-02376-7 (PMC8314446; doi:10.1186/s12877-021-02376-7)
Supplement: Supplementary file 3 — Additional file 3. [file 12877_2021_2376_MOESM3_ESM.docx]

Additional File 3:

**Characteristics of Included Studies**

| **Vestibular Exercises as a Fall Prevention Strategy in Patients with Cognitive Impairment.**  **Varriano.** | |
| --- | --- |
| Study (Year Published) | 2020 |
| Country | Canada |
| Objective/purpose | Assessed the feasibility of recruitment, ability to complete screening tests/questionnaires, feasibility of the program and compliance to a vestibular rehabilitation program in patients with mild cognitive decline, vestibular impairment and previous falls. |
| Study design | Randomized controlled trial. |
| Recruitment setting and/or recruitment methods | Patients who had attended the Memory Clinic and were ≥65 years of age, English speaking, diagnosis of mild cognitive impairment/Alzheimer’s dementia/vascular dementia/mixed dementia, fell within the past year and able to provide consent were contacted. Eligible patients were called (one message/patient) and invited to a screen for vestibular impairment. |
| Study setting and number of sites | N/R |
| Level of analysis | Individual |
| Target population | Patients with mild cognitive decline, vestibular impairment and previous falls. |
| Inclusion Criteria and Exclusion Criteria | Inclusion: Patients who had attended the Memory Clinic and were ≥65 years of age, English speaking, diagnosis of mild cognitive impairment/Alzheimer’s dementia/vascular dementia/mixed dementia, fell within the past year and able to provide consent.  Exclusion: Additional neurological disease (i.e. seizures or stroke), severe psychiatric disease, current inner-ear infection, severe neck arthritis, Montreal cognitive assessment score < 15 or > 26 or other aetiology of dementia (i.e. Parkinson’s disease). |
| Cognitive impairment tool | Diagnosis; Montreal cognitive assessment score between 15 to 26. |
| Baseline cognitive impairment | Overall score (SD): 21.2 (2.9)  Intervention score (SD): N/R  Control score (SD): N/R |
| Total sample n (number eligible) | 48 |
| Intervention n (number invited) | 7 |
| Control n (number invited) | N/R |
| Participation rate | 7/48 = 14.6% |
| Attrition/Loss to follow-up: I n (%); C n (%) | Attrition rate of 71.4% (5/7). Retention in rehabilitation was not feasible (lost 4/5 from intervention, 1/2 from control). |
| Age | Mean age overall (SD): 79.1 (6.7)  Mean age intervention (SD): N/R  Mean age control (SD): N/R |
| Gender: overall n (%); I n (%); C n (%) | Overall: Female: 4 (57), Male: 3 (43) |
| Race/Ethnicity | N/R |
| SES status*(reported by income or education level ONLY)* | N/R |
| Co-morbidities/chronic conditions | N/R |
| Description of intervention | Patients in the vestibular rehabilitation arm were assigned 12 weeks of exercises. Twelve weeks later, patients returned and retested for changes from baseline (activities-specific balance confidence scale, dizziness-handicap inventory, dynamic gait index, World Health Organization Quality of Life BREF and Quality of life Alzheimer’s disease). In addition, patients were given the Problematic Experiences of Therapy Scale. The scale was used to gauge barriers to vestibular rehabilitation adherence. At 24 weeks, patients were called to ask if any falls occurred. Participants in the vestibular rehabilitation arm performed vestibular exercises three times daily, 3–10 minutes/session. Exercises progressed every two weeks. |
| Type of intervention | Exercise; vestibular exercises. |
| Duration of intervention | 12 weeks. |
| Frequency, timing, and duration of intervention contacts/visits | Eight total contacts. Baseline, post-intervention, bi-weekly calls for compliance. Duration of intervention contacts: N/R. |
| Description of control | Patients in the control arm continued with standard care (no exercise program). |
| Follow-up beyond intervention length | At 24 weeks, patients were called to ask if any falls occurred (12 weeks post intervention). |
| Serious adverse events | Falls, but unclear if due to intervention. |
| Funding Source | Krembil Studentship in Dementia Research, Toronto General/Western Hospital Foundation Grant, Unilever Fellowship in Neurosciences, Alzheimer’s Association Travel Fellowship and SGS Travel Grant. |

| **Promoting activity, Independence and stability in early dementia (PrAISED): a, multisite, randomised controlled, feasibility trial. Goldberg.** | |
| --- | --- |
| Study (Year Published) | 2019 |
| Country | United Kingdom |
| Objective/purpose | Aimed to keep people living with early dementia or mild cognitive impairment independent and active, whilst reducing risk of falls, and focused on promoting uptake and adherence. Specific trial feasibility questions were: i) Could participants be recruited and randomised at a sufficient rate (set at >2.5 participants/week from two sites)? ii) Could interventions be delivered across sites and in patient participants’ homes? iii) Is the intervention adhered to and how many withdraw (Set at > 75% of participants retained in the study)? iv) What level of supervision intensity is required that will enable engagement at a level likely to be effective? v) Are there any unexpected or adverse consequences? vi) Could blinded trial data be collected at baseline and follow- up without burdening participants (set at <20% missing primary outcome)? vii) Are sample size assumptions correct? |
| Study design | Randomized controlled trial. |
| Recruitment setting and/or recruitment methods | Patient participants were identified by clinicians working in Memory Assessment Services and referred to the research team if interested, or by researchers through the Join Dementia Research Register. A family member or informal carer of the patient participant who could communicate in English was recruited, if willing. |
| Study setting and number of sites | Home-based, two sites. |
| Level of analysis | Individual |
| Target population | People with mild impairments were selected since the intervention was designed to prevent or slow decline amongst those still well enough to engage and learn new lifestyle activities. |
| Inclusion Criteria and Exclusion Criteria | Inclusion: Age ≥65 years and had diagnosed mild dementia or mild cognitive impairment (Montreal Cognitive Assessment score of 15–25; Mini Mental State Exam score of 18–26 or Addenbrookes Cognitive Examination III score of 60–94, depending on what assessments clinics used). Able to walk without human assistance and communicate in English, have no co-morbidities that prevented participation in cognitive assessment and capacity to give informed consent (formally assessed by a researcher).  Exclusion: N/R |
| Cognitive impairment tool | Montreal Cognitive Assessment (MoCA) score between 15-25; standardised Mini Mental State Exam between 18-26; Addenbrookes Cognitive Examination III score between 60-94. |
| Baseline cognitive impairment | Overall score (SD): standardised Mini Mental State Exam: 25.6 (3.1)  Intervention score (SD): Moderate intensity: 24.8 (3.6), High intensity: 26.2 (3.2)  Control score (SD): 25.9 (2.4) |
| Total sample n (number eligible) | 60 patient participants and 54 carer participants (371 people with mild dementia or mild cognitive impairment were referred to the study and 201 were pre-screened). |
| Intervention n (number invited) | 39 |
| Control n (number invited) | 21 |
| Participation rate | 60/201 = 29.9% |
| Attrition/Loss to follow-up: I n (%); C n (%) | Attrition rate of 18% (11/60).  Moderate intensity: 4 (21), High Intensity: 2 (10); C: 5 (24) |
| Age | Mean age overall (SD): 76 (range 65–91)  Mean age intervention (SD): N/R  Mean age control (SD): N/R |
| Gender: overall n (%); I n (%); C n (%) | Overall: Female: 26 (43); Male: 34 (57)  Female: Moderate intensity: 7 (37), High intensity: 6 (30); 13 (62); C: 13 (62)  Male: Moderate intensity: 12 (63), High intensity: 14 (70); 8 (38); C: 8 (38) |
| Race/Ethnicity | n (%)  White: Overall: 58/60 (97); Moderate intensity: 18 (95); High intensity: 20 (100); Total: 58/60 (97); Control: 20 (95) |
| SES status*(reported by income or education level ONLY)* | Education: n(%) Overall; Moderate intensity; High intensity; Control  Primary school education or less: 0 (0); 1 (5); 0 (0); 1 (2)  Secondary education: 11 (52); 11 (58); 10 (50); 32 (53)  Further education: 9 (43); 7 (37); 10 (50); 26 (43) |
| Co-morbidities/chronic conditions | N/R |
| Description of intervention | Both versions of the PrAISED intervention aimed for the patient participant to complete three hours of PrAISED exercises each week for the 12-month intervention period. Both versions included assessment, creation of an individualised tailored exercise and activity plan, supervised exercises and activity, and regular reassessments and progression but differed in the amount of professional supervision offered. Patient participants in the high intensity supervision group received up to 50 visits from a therapist or rehabilitation support worker over a period of one year. Patient participants in the moderate intensity supervision group received nine visits from a therapist (or two therapists for initial assessment visits) and three phone calls over a three-month period, and encouragement to continue the programme after professional supervision ceased.  The PrAISED physical exercises included balance challenging, strength building, dual-task training and gait re-education. Some exercises could be gained through functional strategies which aimed to maintain or improve independence.  Both interventions included motivational strategies to encourage adherence to, and persistence with, the planned exercises and activities. Participants were encouraged to continue their exercise and activities beyond the supervised period and were provided information on appropriate community exercise groups in their area. |
| Type of intervention | Exercise; balance, strength, dual-task training, gait re-education. |
| Duration of intervention | 12 months. |
| Frequency, timing, and duration of intervention contacts/visits | Moderate intensity: received tapered visits in the first three months of the 12-month intervention period (nine visits and three phone calls, then no contact). High intensity: 50 visits that were tapered over the year, starting at twice weekly for three months, then weekly for three months, bimonthly for three months and monthly for the last three months.  Both groups had baseline and post-int measurement visits.  Mean PrAISED related physical activity minutes/week for both intervention groups was 72 minutes (SD = 63) In the moderate intensity supervision group, participants completed a mean of 77 minutes (SD = 71; range 15 to 228 minutes) per week and 71 minutes (SD = 56; range 11 to 246 minutes) in the high intensity supervision group. |
| Description of control | Single falls prevention assessment and advice with one to two follow-up visits by a therapist if indicated. |
| Follow-up beyond intervention length | N/A |
| Serious adverse events | Adverse events were defined as hospitalisation, or incidents which were life threatening, causing persistent or significant disability or incapacity, or an incident, injury or symptom related to therapy sessions or exercise undertaken independently. There were 19 recorded adverse events. Five were related to the intervention but not serious, 12 were serious but not related, two were neither serious nor related to the intervention. They were all recorded in the active intervention groups but were subject to ascertainment bias as these groups had much more contact with therapists. |
| Funding Source | National Institute for Health Research under its Programme Grants for Applied Research funding scheme (RP-PG-0614- 20007). |

| **Home-Based Exercise Program Improves Balance and Fear of Falling in Community-Dwelling Older Adults with Mild Alzheimer's Disease: A Pilot Study. Padala.** | |
| --- | --- |
| Study (Year Published) | 2017 |
| Country | USA |
| Objective/purpose | Studied the effects of an eight-week home-based caregiver supervised Wii-Fit program on balance in community-dwelling older adults with mild Alzheimer’s disease. Explored the effects of the exercise program on fear of falling, functional state, quality of life, and cognition in the same subjects. |
| Study design | Randomized controlled trial. |
| Recruitment setting and/or recruitment methods | The medical center’s electronic medical records were reviewed for pre-screening. Recruits that met the pre-screening criteria by the electronic medical record review were invited for the baseline visit. Recruitment was done at the medical center. |
| Study setting and number of sites | Home-based. N/A |
| Level of analysis | Individual. |
| Target population | Alzheimer's disease patients. |
| Inclusion Criteria and Exclusion Criteria | Inclusion: Community-dwelling older adults ≥60 years with Alzheimer’s Disease (Diagnostic and Statistical Manual DSM-IV TR criteria), a Mini Mental State Exam score ≥18, with history or fear of falling in the past year and had a caregiver.  Exclusion: Individuals using wheel chairs or walkers for mobility or having absolute contraindications to exercise per American College of Sports Medicine guideline. |
| Cognitive impairment tool | Diagnosis; Diagnostic and Statistical Manual DSM-IV TR criteria; Mini Mental State Exam score ≥ 18 |
| Baseline cognitive impairment | Overall Mini Mental State Exam score (SD): 22.9 (2.2)  Intervention Mini Mental State Exam score (SD): 23.3 (2.2)  Control Mini Mental State Exam score (SD): 22.7 (2.3) |
| Total sample n (number eligible) | 30 (106 screened; 57 did not meet inclusion criteria, 19 declined to participate). |
| Intervention n (number invited) | 15 |
| Control n (number invited) | 15 |
| Participation rate | 30/106 = 28.3% |
| Attrition/Loss to follow-up: I n (%); C n (%) | Attrition rate of 20% (6/30).  I: 3 (20); C: 3 (20) |
| Age | Mean age overall (SD): 73.0 (6.2)  Mean age intervention (SD): 72.1 (5.3)  Mean age control (SD): 73.9 (7.1) |
| Gender: overall n (%); I n (%); C n (%) | Overall: Female: 11 (37), Male: 19 (63)  Female: I: 5 (33); 6 (40)  Male: I: 10 (67); C: 9 (60) |
| Race/Ethnicity | n (%) Overall; I; C  Non-Hispanic Caucasian: 23 (76.7); 10 (66.7); 13 (86.7)  Non-Hispanic African-American: 7 (23.3); 5 (33.3); 2 (13.3) |
| SES status*(reported by income or education level ONLY)* | n (%) Overall; I; C  High school diploma: 26 (87); 13 (87); 13 (87) Some college: 2 (7); 1 (7); 1 (7)  Bachelor’s degree: 2 (7); 1 (7); 1 (7) |
| Co-morbidities/chronic conditions | n (%) Overall; I; C  Hypertension: 28 (93); 13 (87); 15 (100)  Diabetes: 8 (27); 4 (27); 4 (27)  Hyperlipidemia: 27 (90); 13 (87); 14 (93)  Coronary Artery Disease: 8 (27); 4 (27); 4 (27)  Degenerative joint disease: 5 (17); 3 (20); 2 (13)  Depression: 17 (57); 8 (53); 9 (60)  Number of comorbidities, median (IQR): 7 (6, 8); 6 (6, 8); 7 (6, 8) |
| Description of intervention | Both groups exercised under caregiver supervision for 30 minutes five days/week for eight weeks. The Wii-Fit group performed exercises from five categories of the Wii-Fit program: yoga, strength training, aerobics, balance games, and training plus, which included more complex exercise tasks. Each session in the Wii-Fit included a warm up, exercise, and cool down phase. During the warm up and cool down phases, subjects walked for five minutes at a self-selected comfortable pace using the program’s “basic walk” activity.  The exercise phase was designed to be participant centric. The study physician set up the components of the exercise program based on knowledge from the authors’ prior studies of which exercises could be completed by this population with respect to ability and safety. Exercises were deliberately chosen from the balance, aerobic, strength training, and yoga components. The specific exercises in each category were picked based on their ease of use and subject preference expressed in the authors’ clinical work.  All subjects started each exercise at level one. Upon mastery, subsequent levels were opened automatically by the program. Subjects were encouraged to choose one or more exercises from every Wii-Fit category during each session. Research assistants did an initial home visit to set up the program at the patient’s home and trained the subject and their caregiver on the use of the program. Caregivers were instructed to help the subjects in setting up the instruments if needed and supervise them during each exercise session. They were also instructed to help the subjects record the activities performed daily in their activity diary. |
| Type of intervention | Exercise; Wii-fit (yoga, strength, aerobics, balance). |
| Duration of intervention | Eight weeks. |
| Frequency, timing, and duration of intervention contacts/visits | Six contacts. A research assistant made home visits to the Wii-Fit group to set up the device and trained the subject and their caregiver about its use. The research assistants followed up with phone calls one day after installation, one week after starting the program, and then every two weeks to troubleshoot problems, provide encouragement, and identify any adverse events. All outcomes were measured at baseline, eight weeks (end of intervention), and at 16 weeks (eight weeks post intervention) by an outcomes assessor. |
| Description of control | Subjects in the walking program were instructed to walk for 30 minutes at their self-selected pace either indoors or outdoors for five days/week. Subjects were advised to walk in an uninterrupted block of activity and not cumulative over the day. Subjects maintained an activity diary with the assistance from the caregivers wherein they recorded the day and time spent exercising during each session. |
| Follow-up beyond intervention length | Eight weeks after intervention. |
| Serious adverse events | There were four adverse events, but they were deemed to be not study related. |
| Funding Source | New Investigator grant from Alzheimer’s Association. |

| **Falls and Physical Activity in Persons With Mild to Moderate Dementia Participating in an Intensive Motor Training Randomized Controlled Trial. Zieschang.** | |
| --- | --- |
| Study (Year Published) | 2017 |
| Country | Germany |
| Objective/purpose | Analyzed falls data obtained prospectively on motor training in older people with mild to moderate dementia. The intensive, progressive motor training was feasible, safe, and highly effective for lower-limb strength and functional performance. |
| Study design | Randomized controlled trial. |
| Recruitment setting and/or recruitment methods | Patients were recruited either consecutively from rehabilitation wards of a geriatric hospital (AGAPLESION Bethanien Hospital/Centre for Geriatric Medicine at the University of Heidelberg) at the end of rehabilitation, or from outpatient nursing care services from 2006 to 2008. |
| Study setting and number of sites | N/R |
| Level of analysis | Individual |
| Target population | Individuals with dementia who, in comparison, have an increased risk for falls and for adverse outcomes when sustaining a fall. Older people with mild to moderate dementia. |
| Inclusion Criteria and Exclusion Criteria | Inclusion: A dementia diagnosis in accordance with international standards was confirmed in patients who met the screening criteria for cognitive impairment (Mini Mental State Exam scores 17 to 26).  Exclusion: N/R |
| Cognitive impairment tool | Diagnosis (international standards); Mini Mental State Exam score between 17-26 |
| Baseline cognitive impairment | Overall score (SD): N/R  Intervention score (SD): 21.6 (2.9)  Control score (SD): 21.9 (3.3) |
| Total sample n (number eligible) | 122 (1617 of 1961 did not meet inclusion criteria). |
| Intervention n (number invited) | 62 |
| Control n (number invited) | 60 |
| Participation rate | 122/1961 = 6.2% |
| Attrition/Loss to follow-up: I n (%); C n (%) | Attrition rate of 9.8% (12/122)  I: 7 (11); C: 5 (8) |
| Age | Mean age overall (SD): N/R  Mean age intervention (SD): 82.1 (6.6)  Mean age control (SD): 82.2 (6.7) |
| Gender: overall n (%); I n (%); C n (%) | Overall: Female: 81 (74); Male: 29 (26)  Female: I: 40 (73); C: 41 (75)  Male: I: 15 (27); C: 14 (25) |
| Race/Ethnicity | N/R |
| SES status*(reported by income or education level ONLY)* | Years of education [median (range)]: I: 11.0 (7-19); C: 11.0 (7-19) |
| Co-morbidities/chronic conditions | Cumulative Illness Rating Scale, mean (SD): I: 24.2 (3.2); C: 23.7 (3.5) |
| Description of intervention | The duration of the progressive resistance and functional training program was three months (two hours, twice weekly). Resistance training in groups of four to six patients, supervised by a qualified instructor, targeted functionally relevant muscle groups at a submaximal intensity (60% to 80% of the one-repetition maximum). As training progressed, the applied weight was continuously increased. The functional training focused on basic activities of daily living– related motor functions such as keeping balance while standing, walking, stepping, sitting down, and standing up, progressing to advanced levels such as climbing stairs, crossing obstacles on the floor, walking over foam surfaces to challenge the participants’ balance system, and walking with additional cognitive or motor tasks. The training was adapted to the target sample of cognitively impaired patients and was individually adjusted for age and illness-related deficits to prevent overexertion. |
| Type of intervention | Exercise; progressive resistance and functional training (activities of daily living, balance, walking, gait). |
| Duration of intervention | Three months. |
| Frequency, timing, and duration of intervention contacts/visits | Approximately 24 (twice weekly for three months) as well as two measurements (baseline and post intervention) and three telephone reminders.  Intervention group had training for a duration of three months (two hours, twice weekly). Control group had training for one hour, twice weekly. Physical activity was assessed: before randomization; at the end of the three-month training period; at the short-term follow-up three months later; and at the late-term follow-up nine months after training cessation. Telephone reminders monthly (duration of calls were N/R). |
| Description of control | Met twice weekly for a one-hour motor placebo group training supervised by the same qualified instructors. Typical activities were flexibility exercises, calisthenics, low-intensity training with hand-held weights, and ball games while seated. |
| Follow-up beyond intervention length | Short-term follow-up: Three months after training cessation  Long-term follow-up: Nine months after training cessation |
| Serious adverse events | N/R |
| Funding Source | Baden-Württemberg Stiftung, the Robert Bosch Stiftung, the Dietmar Hopp Stiftung, and the Wilhelm Woort Stiftung. |

| **Effects of Tai Chi on Cognition and Fall Risk in Older Adults with Mild Cognitive Impairment: A Randomized Controlled Trial. Sungkarat.** | |
| --- | --- |
| Study (Year Published) | 2017 |
| Country | Thailand |
| Objective/purpose | Investigated whether a program that allowed older adults to learn Tai Chi in a group setting and then continue their exercise at home could improve both specific cognitive abilities and reduce physiological fall risk in older adults with multiple-domain amnestic mild cognitive impairment in a randomized controlled trial. |
| Study design | Randomized controlled trial. |
| Recruitment setting and/or recruitment methods | Participants were recruited from the local community. |
| Study setting and number of sites | Community centre and home-based. One site. |
| Level of analysis | Individual |
| Target population | Older adults with multiple-domain amnestic mild cognitive impairment. The amnestic subtype of mild cognitive impairment is common and is a prodromal symptom of dementia, particularly Alzheimer’s disease. When only memory is impaired, it is classified as single-domain amnestic mild cognitive impairment, while multiple-domain amnestic mild cognitive impairment includes impaired memory and other cognitive domains such as attention, executive function, visuospatial ability, and language are impaired. |
| Inclusion Criteria and Exclusion Criteria | Inclusion: Met Petersen’s criteria for diagnosing amnestic multiple-domain mild cognitive impairment, Mini Mental State Examination score ≥ 24 and Montreal Cognitive Assessment <26. Had adequate memory if cued, and comprehended instructions required for study participation.  Exclusion: medications for cognition, neurological conditions (e.g., Parkinson’s disease, stroke, multiple sclerosis), depressive symptoms, acute or chronic conditions that would preclude exercise, regular exercise (≥30 minutes/day, ≥three days/week). |
| Cognitive impairment tool | Diagnosis (Petersen's criteria); Montreal Cognitive Assessment score <26; Mini Mental State Examination ≥24. |
| Baseline cognitive impairment | Overall score (SD): N/R  Intervention score (SD): Montreal Cognitive Assessment score: 21.2 (3.4); Mini Mental State Examination score: 26.5 (1.7)  Control score (SD): Montreal Cognitive Assessment score: 20.4 (3.8); Mini Mental State Examination score: 25.8 (2.3) |
| Total sample n (number eligible) | 66 (127 of 212 were ineligible). |
| Intervention n (number invited) | 33 |
| Control n (number invited) | 33 |
| Participation rate | 66/212 = 31.1% |
| Attrition/Loss to follow-up: I n (%); C n (%) | Attrition rate of 10.6% (7/66).  I: 3 (9); C: 4 (12) |
| Age | Mean age overall (SD): N/R  Mean age intervention (SD): 68.3 (6.7)  Mean age control (SD): 67.5 (7.3) |
| Gender: overall n (%); I n (%); C n (%) | Overall: Female: 33 (50); Male: 33 (50)  Female: I: 31 (94); C: 26 (79)  Male: I: 2 (6); C: 7 (21) |
| Race/Ethnicity | N/R |
| SES status*(reported by income or education level ONLY)* | Education, years, mean (SD): I: 11.4 (5.1); C: 9.3 (5.5) |
| Co-morbidities/chronic conditions | N/R |
| Description of intervention | Participants in the Tai Chi group attended Tai Chi classes led by a certified Tai Chi instructor for three weeks (nine sessions) to learn Tai Chi principles and the 10-form Tai Chi. The Tai Chi classes were held with six or seven persons/class in the exercise room at the Department of Physical Therapy. Participants then practiced Tai Chi at home three times/week for 12 weeks. A 50-minute video was provided to assist participants with their Tai Chi home exercises. Each 50-minute session included a 10-minute warm-up (range of motion, stretching), 30 minutes of Tai Chi exercise, and a 10-minute cool-down (stretching, breathing exercises). For the 30 minutes of Tai Chi exercise, participants practiced the 10-form Tai Chi similar to the program used in a previous study. Participants were asked to fill in a logbook immediately after their exercise sessions to maximize the accuracy of information. Several additional strategies were used to assist with exercise adherence, including requesting family members to remind participants about their exercise program and placing the exercise schedule in a prominent location in the home. Research staff also made weekly recorded reminder telephone calls, and information gained from these conversations about exercise participation was cross-validated with the logbook entries. Participants in both groups were asked to maintain their routine lifestyle throughout the study and to inform the research team if they changed their routines or health events occurred. All participants also received a telephone call from the research staff once a week to monitor Tai Chi training frequency, duration, and adverse events (Tai Chi group) and any health and lifestyle changes (e.g., attending a recreation club, medication and supplement use, illnesses and hospital admissions). |
| Type of intervention | Exercise; Tai Chi. |
| Duration of intervention | 15 weeks. |
| Frequency, timing, and duration of intervention contacts/visits | 45 sessions as well as 12 weekly phone calls and two measurement visits (baseline and end of week 15).  Tai Chi classes led by a certified Tai Chi instructor for three weeks (nine sessions). Three weeks center-based and 12 weeks home-based Tai Chi (50 minutes/session, three times/week). Duration of calls and measurements were N/R. |
| Description of control | Participants in the control group received educational material covering information related to cognitive impairment and fall prevention. |
| Follow-up beyond intervention length | N/A |
| Serious adverse events | No adverse events found. |
| Funding Source | Thailand Research Fund, RSA5680020 and a Research Chair Grant from the National Science and Technology Development Agency Thailand. |

| **Sensor-based balance training with motion feedback in people with mild cognitive impairment.**  **Schwenk.** | |
| --- | --- |
| Study (Year Published) | 2016 |
| Country | USA |
| Objective/purpose | Evaluated the feasibility and experience of a four-week sensor-based training program to measure the effects on balance in a sample of memory clinic patients with clinically confirmed amnestic mild cognitive impairment. |
| Study design | Randomized controlled trial. |
| Recruitment setting and/or recruitment methods | Individuals were recruited at the Cleo Roberts Memory and Movement Disorders Center of the Banner Sun Health Research Institute (Sun City, Arizona). Recruitment started in July 2014, and follow-up was completed in September 2014. |
| Study setting and number of sites | Research centre. One site |
| Level of analysis | Individual |
| Target population | Individuals with clinically confirmed amnestic mild cognitive impairment. |
| Inclusion Criteria and Exclusion Criteria | Inclusion: Community-dwelling outpatients with confirmed diagnosis of amnestic mild cognitive impairment according to international established criteria.  Exclusion: severe cognitive impairment (Montreal Cognitive Assessment [MOCA] score <20, non-ambulatory or major mobility disorder, other neurological conditions associated with cognitive impairment such as: stroke; Parkinson disease; and head injury. Any clinically significant psychiatric condition, current drug or alcohol abuse, or laboratory abnormality that would interfere with the ability to participate in the study. Severe visual impairment. Unwillingness to participate. |
| Cognitive impairment tool | Diagnosis (Petersen's criteria); Montreal Cognitive Assessment score >20 |
| Baseline cognitive impairment | Overall score (SD): 23.3 (2.6)  Intervention score (SD): 23.3 (3.1)  Control score (SD): 22.4 (3.0) |
| Total sample n (number eligible) | 22 (15 of 37 declined to participate). |
| Intervention n (number invited) | 12 |
| Control n (number invited) | 10 |
| Participation rate | 22/37 = 59.5% |
| Attrition/Loss to follow-up: I n (%); C n (%) | Attrition rate of 9.1% (2/22).  I: 1 (8); C: 1 (10) |
| Age | Mean age overall (SD): 78.2 (8.7)  Mean age intervention (SD): 77.8 (6.9)  Mean age control (SD): 79.0 (10.4) |
| Gender: overall n (%); I n (%); C n (%) | Overall: Female: 12 (55); Male: 10 (45)  Female: I: 7 (58); C: 5 (50)  Male: I: 5 (42); C: 5 (50) |
| Race/Ethnicity | N/R |
| SES status*(reported by income or education level ONLY)* | Education, years (SD): I: 14.2 ± 2.3; C: 15.9 ± 2.7 |
| Co-morbidities/chronic conditions | Diagnoses, No (SD): I: 2.5 (1.6); C: 3.5 (2.1)  Prescriptions, No (SD): I: 4.0 (2.0); CON 6.3 (3.9) |
| Description of intervention | The technology used in this study consisted of a 24-inch computer screen, an interactive virtual user interface, and five inertial sensors including a triaxial accelerometer, gyroscope, and magnetometer for estimation of joint angles and position. Sensor data were acquired and transmitted at a 100Hz frequency for real-time feedback in a virtual environment. The sensors were mounted in two places (the upper and lower leg) on both legs and on the lower back using elastic straps. Training was conducted in a separate room in the Cleo Roberts Memory and Movement Disorders Center. The participant stood in front of the screen, which was positioned at eye level. A chair with a backrest was in front of the participant to provide support if required. A supervisor gave instructions about the exercise tasks during the first training session. In subsequent sessions, subjects conducted exercises based on sensor feedback only; however, the supervisor remained with the participant during all sessions to guarantee safety. Participants attended two training sessions/week for four weeks. Sessions lasted approximately 45 minutes and included ankle point-to-point reaching tasks and virtual obstacle-crossing tasks. Frequency and duration were determined based on the authors’ previous study in cognitively intact older adults. The interface was designed to be intuitive and easy to navigate and to avoid complex animations that could distract cognitively impaired persons from observing relevant information related to motion performance and motor error. |
| Type of intervention | Exercise; Balance (ankle point-to-point reaching tasks and virtual obstacle-crossing tasks). |
| Duration of intervention | Four weeks. |
| Frequency, timing, and duration of intervention contacts/visits | Eight training sessions and two measurements. Two training sessions/week for four weeks. The duration of each training session was 45 minutes; measurements N/R. |
| Description of control | No training. |
| Follow-up beyond intervention length | N/A |
| Serious adverse events | No training-related adverse events occurred. |
| Funding Source | Flinn Foundation (award 1907), the National Institute on Aging (Center Core Grant award P30 AG019610), and the National Institutes of Health (SBIR award number 1R43AG044882–01A1). |

| **Donepezil for gait and falls in mild cognitive impairment: a randomized controlled trial.**  **Montero-Odasso.** | |
| --- | --- |
| Study (Year Published) | 2019 |
| Country | Canada |
| Objective/purpose | Assessed whether donepezil could improve gait performance and dual-task gait cost in older individuals with mild cognitive impairment. Secondary aims included improvement in cognition and reduction of fall rate. |
| Study design | Randomized controlled trial. |
| Recruitment setting and/or recruitment methods | Recruitment occurred from June 2009 to November 2015 from memory clinics at Parkwood Institute (London, Ontario, Canada) and the Jewish General Hospital. |
| Study setting and number of sites | Home-based. |
| Level of analysis | Individual. |
| Target population | Older adults with mild cognitive impairment, an intermediate state between normal cognitive aging and early dementia. |
| Inclusion Criteria and Exclusion Criteria | Inclusion: Age ≥65 years, able to walk 10m without a gait aid and had mild cognitive impairment, ascertained by scoring 0.5 on the global rating of the Clinical Dementia Rating scale and satisfying Winblad’s criteria.  Exclusion: lack of English proficiency, parkinsonism or any neurological or musculoskeletal disorder with residual motor deficits (e.g. stroke, epilepsy), low body weight (<45 kg), possible diagnosis of Alzheimer’s disease, use of herbal preparations (St John’s Wort and Gingko biloba), history of substance abuse, use of anticholinergic agents (e.g. benztropines), use of other acetylcholinesterase inhibitors or cholinergic agents (e.g. bethanechol), major depression (8/15 on the Geriatric Depression Scale), history of liver diseases (hepatitis or cirrhosis), bradycardia or sick-sinus syndrome, previous intolerance/allergy to donepezil, severe chronic obstructive pulmonary disease and/or asthma and history of seizures. |
| Cognitive impairment tool | Clinical Dementia Rating scale score 0.5; Winblad's criteria (Petersen); standardized Mini Mental State Examination; Montreal Cognitive Assessment |
| Baseline cognitive impairment | Overall score (SD): standardized Mini Mental State Examination: 27.47 (1.96), Montreal Cognitive Assessment: 23.60 (2.52)  Intervention score (SD): standardized Mini Mental State Examination: 27.42 (2.19), Montreal Cognitive Assessment: 23.19 (2.55)  Control score (SD): standardized Mini Mental State Examination: 27.52 (1.72), Montreal Cognitive Assessment: 22.97 (2.37) |
| Total sample n (number eligible) | 60 (98 of 262 were ineligible, 104 of 262 refused to participate). |
| Intervention n (number invited) | 31 |
| Control n (number invited) | 29 |
| Participation rate | 60/262 = 22.9% |
| Attrition/Loss to follow-up: I n (%); C n (%) | Attrition rate of 25% (15/60)  I: 11 (35); C: 4 (14) |
| Age | Mean age overall (SD): 75.28 (7.18)  Mean age intervention (SD): 73.45 (5.74)  Mean age control (SD): 77.24 (8.11) |
| Gender: overall n (%); I n (%); C n (%) | Overall: Female: 27 (45); Male: 33 (55)  Female: I: 15 (48); C: 12 (41)  Male: I: 16 (52); C: 17 (59) |
| Race/Ethnicity | N/R |
| SES status*(reported by income or education level ONLY)* | N/R |
| Co-morbidities/chronic conditions | No. of comorbidities, mean (SD): 5.60 (3.03) 5.07 (3.08) 6.10 (2.94) n (%) Overall; I; C  Hypertension: 32 (53); 20 (41); 12 (41)  Osteoporosis: 6 (10); 1 (3); 5 (17) Osteoarthritis: 13 (22); 8 (26); 5 (17) Dyslipidemia: 30 (50); 20 (64); 10 (34) Stroke: 2 (3); 1 (3); 1 (3) Myocardial infarct: 4 (7); 2 (6); 2 (7) Atrial fibrillation: 1 (2); 1 (3); 0 Amnestic mild cognitive impairment: 40 (67); 20 (65); 20 (69) Multiple-domain mild cognitive impairment: 17 (28); 7 (22); 10 (34) |
| Description of intervention | After informed consents were signed, participants underwent baseline, one-month and six-month assessments. Baseline gait assessment occurred within one week prior to starting donepezil or matching placebo and the one-month assessment occurred after four weeks of treatment at 5mg/day of donepezil or matching placebo. Donepezil was increased to the full dose of 10 mg/day after four weeks. Final assessment occurred after five months of receiving full dose of donepezil or placebo. Donepezil and placebo were provided by Pfizer Canada Inc. to assure perfect matching and administrated in kits based on randomization sequence. Assessments at each time-point performed by blind assessors included gait analysis, cognitive testing and fall incidence information. |
| Type of intervention | Medication or vitamin supplement; Donepezil |
| Duration of intervention | Six months. |
| Frequency, timing, and duration of intervention contacts/visits | Three assessments, four monthly phone calls [Baseline (T0), one-month (T1) and six-month (T6) assessments phone calls (T2, T3, T4 and T5)]. Duration of contacts N/R. |
| Description of control | Placebo. |
| Follow-up beyond intervention length | N/A |
| Serious adverse events | The adverse events frequency (22%) was higher but the proportion of participants discontinuing treatment (25%) was lower than that reported in a larger clinical trial using donepezil in mild cognitive impairment. Most adverse events were considered to be mild (80%) and required no treatment. No major adverse events were reported. |
| Funding Source | Physician Services Incorporated Foundation of Canada. |

| **Musical dual-task training in patients with mild-to-moderate dementia: a randomized controlled trial. Chen.** | |
| --- | --- |
| Study (Year Published) | 2018 |
| Country | Taiwan |
| Objective/purpose | Investigated the effects of musical dual-task training on attention control,  dual-task performance, balance, falls efficacy, and agitation in older adults with mild-to-moderate dementia. |
| Study design | Randomized controlled trial. |
| Recruitment setting and/or recruitment methods | Participants were recruited via referrals by neurologists from a specialized dementia outpatient unit at a medical center and by case managers in cooperation with neurologists at a city district health center in Taiwan from October 2012 to March 2013. |
| Study setting and number of sites | Community centre/research facility. Number of sites N/R. |
| Level of analysis | Individual |
| Target population | Patients with dementia are more prone to incidental falls, they have higher fracture rates than age-matched people with intact cognition. Compared with normal subjects, patients with dementia have been found to walk with a slower speed and higher stride time variability when asked to walk while counting backwards or naming as many words as possible starting with a specific letter. |
| Inclusion Criteria and Exclusion Criteria | Inclusion: Age ≥ 55 years, mild-to-moderate dementia with a Clinical Dementia Rating score of 0.5, 1, or 2, as confirmed by a certified neurologist, independently walk 10m independently without using a walker, cane, or receiving assistance from another person.  Exclusion: had other known neurological disorders, such as acute stroke or Parkinson’s disease, or significant orthopedic, visual, or hearing impairments that hindered ambulation. |
| Cognitive impairment tool | Mini Mental State Examination. Clinical Dementia Rating scale score 0.5, 1, or 2; confirmed by neurologist. |
| Baseline cognitive impairment | Overall score (SD): N/R  Intervention score (SD): Mini Mental State Examination score: 16.4 (7.3). Clinical Dementia Rating scale score (score = n): 0.5 = 6, 1.0 = 6, 2.0 = 3.  Control score (SD): Mini Mental State Examination score: 17.9 (3.7). Clinical Dementia Rating scale score (score = n): 0.5 = 3, 1.0 = 9, 2.0 = 1 |
| Total sample n (number eligible) | 30 (32 were eligible). |
| Intervention n (number invited) | 15 |
| Control n (number invited) | 15 |
| Participation rate | 30/32 = 93.8% |
| Attrition/Loss to follow-up: I n (%); C n (%) | Attrition rate of 6.7% (2/30).  I: 0; C: 2 (13) |
| Age | Mean age overall (SD): N/R  Mean age intervention (SD): 77.3 (9.4)  Mean age control (SD): 77.3 (10.0) |
| Gender: overall n (%); I n (%); C n (%) | Overall: Female: 14 (50); Male: 14 (50)  Female: I: 9 (64); C: 5 (36)  Male: I: 6 (43); C: 8 (57) |
| Race/Ethnicity | N/R |
| SES status*(reported by income or education level ONLY)* | N/R |
| Co-morbidities/chronic conditions | N/R |
| Description of intervention | The protocol of the musical dual-task training group was designed to train participants in scenarios analogous to natural situations. The participants were asked to respond to obstacles (visual stimuli) and engage in conversation (auditory stimuli) while walking. The protocol also included a musical task and a walking task. The musical task comprised two types of activities: singing and playing simple percussive musical instruments. Each of these activities had two versions with different attention levels. For the music task, the participants made music autonomously using their own self-generated rhythm in one version (whether playing a steady beat or playing freely) and received visual or auditory cues from the therapist in the other version. In the walking task, the participants either walked forward or stepped sideways. Between the musical task and walking task, there were eight possible combinations of dual-task activities, ranging from singing familiar songs and walking (lowest cognitive load) to playing an instrument contingently and side-stepping (highest cognitive load). Thus, the therapist presented the participants with different challenges with varying cognitive loading to enhance the participant’s ability. The participants were instructed to perform all eight combinations progressively within a session. The music was selected according to the preferred songs of the individual participants and included well-known folk songs and songs popular during the time period in which the participants were young adults. Individual music preferences were obtained from interviews with the participants and their guardians/caregivers. The music presented in the sessions was performed live by the music therapist who sang and played the guitar or keyboard. The live performance cued the patient to respond and provided temporal cues to facilitate walking movements. |
| Type of intervention | Multifactorial; Musical dual-task training (physical and cognitive tasks). |
| Duration of intervention | Two months. |
| Frequency, timing, and duration of intervention contacts/visits | Two measurement visits, eight weekly individual 60-minute sessions. Individuals in both groups were assessed approximately one week before and one week after training sessions. Duration of measurements N/R. |
| Description of control | Participated in activities involving non-musical cognitive tasks and walking exercises. The cognitive tasks were determined according to the preferences of the individual participants, and included chess, playing cards, reading, writing, completing mathematical exercises, puzzles, and games. During each control session, the music therapist alternately conducted cognitive tasks and took walks with the participant. Each participant took three-to-five walks across the room accompanied by the music therapist, depending on their physical condition that day. Between walks, participants sat and rested for one–two minutes. |
| Follow-up beyond intervention length | N/A |
| Serious adverse events | No adverse events such as falls or emotional distress occurred in both groups. The primary investigator was experienced in adjusting the pace and in reacting to the responses of the participants to alleviate potential distress induced by dual-tasking. In addition, many participants were not necessarily aware of their dual-task performance. They were not told whether or not they were doing anything “wrong”. They simply continued practicing. |
| Funding Source | Ministry of Education, Taiwan. |

| **The effects of occupation-centered activity program on fall-related factors and quality of life in patients with dementia. Kim.** | |
| --- | --- |
| Study (Year Published) | 2017 |
| Country | Korea |
| Objective/purpose | Developed and implemented an occupation-centered activity program that contains activities specific to dementia, examined the efficacy of the occupation-centered activity program in regard to fall-related factors and quality of life of the patients, and contributed to improving the design of future programs targeted for providing therapy for dementia patients to alleviate their symptoms. |
| Study design | Clinical controlled trial. |
| Recruitment setting and/or recruitment methods | N/R |
| Study setting and number of sites | Community care centre. One site. |
| Level of analysis | Individual |
| Target population | Older individuals with dementia. |
| Inclusion Criteria and Exclusion Criteria | Inclusion: dementia patients admitted to daytime care center in Jeolla Province. All of the subjects were diagnosed with dementia by neuropsychiatrists.  Exclusion: N/R |
| Cognitive impairment tool | Diagnosis; Alzheimer's dementia or vascular dementia. Mini Mental State Examination-Korea. |
| Baseline cognitive impairment | Overall score (SD): N/R  Intervention score (SD): Mini Mental State Examination-Korea: 15.5 (2.9)  Control score (SD): Mini Mental State Examination-Korea: 15.6 (2.4) |
| Total sample n (number eligible) | 30 (number eligible N/R) |
| Intervention n (number invited) | 15 |
| Control n (number invited) | 15 |
| Participation rate | N/R |
| Attrition/Loss to follow-up: I n (%); C n (%) | N/R |
| Age | Mean age overall (SD): N/R  Mean age intervention (SD): 82.0 (4.6)  Mean age control (SD): 80.9 (3.4) |
| Gender: overall n (%); I n (%); C n (%) | Overall: Female: 6 (20); Male: 24 (80)  Female: I: 2 (13) C: 4 (27)  Male: I: 13 (87); 11 (73) |
| Race/Ethnicity | N/R |
| SES status*(reported by income or education level ONLY)* | Education years, n (%) I; C: 0 years: 7 (47); 8 (53) 6 years: 4 (27); 2 (13) 9 years: 3 (20); 5 (33) 12 years: 1 (7); 0 (0) |
| Co-morbidities/chronic conditions | N/R |
| Description of intervention | Participated in the occupation-centered activity program and took medications for their dementia symptoms. This study applied the program five times/week for a total of 24 sessions for approximately 60 minutes/ session. The program included physical activities, cognitive activities, daily life activities, instrumental daily life activities, handicraft, traditional Korean music activities, and other music activities. This program included calibrated activities appropriate and meaningful for dementia patients. |
| Type of intervention | Multifactorial; physical activities, cognitive activities, activities of daily living, music activities. |
| Duration of intervention | 12 weeks. |
| Frequency, timing, and duration of intervention contacts/visits | 24 sessions and two measurements (pre and post). The program was performed five times/week for a total of 24 sessions for approximately 60 minutes/session. Duration of measurements N/R. |
| Description of control | Did not participate in the occupation-centered activity program but continued medications for their dementia symptoms. |
| Follow-up beyond intervention length | N/A |
| Serious adverse events | N/R |
| Funding Source | N/R |

| **A feasibility study and pilot randomised trial of a tailored prevention program to reduce falls in older people with mild dementia. Wesson.** | |
| --- | --- |
| Study (Year Published) | 2013 |
| Country | Australia |
| Objective/purpose | Tested the feasibility of the study components and the acceptability of a home safety and exercise fall prevention program for people with mild dementia and implemented as a carer-supported intervention. |
| Study design | Randomized controlled trial. |
| Recruitment setting and/or recruitment methods | A convenience sample of dyads of people with mild dementia and their carers was recruited from a Memory Disorders, a Cognitive Disorders and an Aged Care Clinic, and a clinical dementia service network within the local health network in the eastern suburbs of Sydney, Australia. Recruitment method: referral. |
| Study setting and number of sites | Home-based. Number of sites N/A. |
| Level of analysis | Individual |
| Target population | Older adults living with dementia. |
| Inclusion Criteria and Exclusion Criteria | Inclusion: Community-dwelling people > 65 years with a specialist diagnosis of dementia or an Addenbrooke’s Cognitive Examination score of ≤82 indicating the cut off score for dementia. Participants also had a non-paid carer (usually a family member) with a minimum of 3.5 hours/week of face to face contact. Carer participation was essential for the delivery of the intervention and for assisting with the recall of falls. Participants had to be English speaking given that all assessments and interventions were conducted in English.  Exclusion: delirium or an acute medical condition; severe psychiatric disorder or progressive neurological disorder (except dementia); a Mini Mental State Exam < 12 (given the likely difficulties of following simple commands); severe visual impairment (as visual cues were utilised to enhance uptake of exercises); and residents of aged care facilities. |
| Cognitive impairment tool | Diagnosis; Addenbrooke’s cognitive examination – revised score of ≤ 82 (score 0-100; cut off for dementia: 82). Mini Mental State Exam. |
| Baseline cognitive impairment | Overall score (SD): N/R  Intervention score (SD): Addenbrooke’s cognitive examination – revised score: 67.8 (12.6), Mini Mental State Exam score: 24.5 (3.1)  Control score (SD): Addenbrooke’s cognitive examination – revised score: 62.5 (14.2), Mini Mental State Exam score: 22.5 (4.3) |
| Total sample n (number eligible) | 22 (Eight of 38 were ineligible). |
| Intervention n (number invited) | 11 |
| Control n (number invited) | 11 |
| Participation rate | 22/38 = 57.9% |
| Attrition/Loss to follow-up: I n (%); C n (%) | Attrition rate of 4.5% (1/22).  I: 1 (9); C: 0 |
| Age | Mean age overall (SD): N/R  Mean age intervention (SD): 78.7 (4.2)  Mean age control (SD): 80.9 (5.0) |
| Gender: overall n (%); I n (%); C n (%) | Overall: Female: 9 (41); Male: 13 (59)  Female: I: 5 (46); C: 4 (36)  Male: I: 6 (54); C: 7 (64) |
| Race/Ethnicity | N/R |
| SES status*(reported by income or education level ONLY)* | Years of education, mean (SD):  I: 10.6 (2.4); C: 12.0 (4.3) |
| Co-morbidities/chronic conditions | Number of co-morbidities, mean (SD):  I: 3.0 (2.4); C: 4.1 (2.2) |
| Description of intervention | Conducted in participants’ homes and consisted of strength and balance training exercises as well as home hazard reduction. The Westmead Home Safety Assessment was used as a tool by the occupational therapist, the carer and the person with dementia, to audit the home environment systematically for environmental and behavioural fall hazards. Participants were provided with a booklet of home safety recommendations which formed the basis of subsequent occupational therapy visits. Recommendations were tailored to the specific hazards identified in participants’ homes and the format was adapted to cognitive abilities. Some smaller items, such as sensor lights were provided by the occupational therapy investigator and trialled before participants purchased them. Each participant was prescribed up to six individually tailored strength and balance exercises which were selected from the Weight-Bearing Exercise for Better Balance program and based on the results of the physical performance assessment. Strength training exercises included sit to stand, calf raises and step ups onto a block. Static balance tasks included a series of stance positions with diminishing base of support (i.e. standing with feet together, semi tandem, near tandem and tandem) with eyes open or closed. Dynamic balance exercises included stepping over a strip of matting on the floor, foot taps onto a block, lateral side steps, sideways walking and step ups. Possible progression included increased frequency, increased repetitions, decreased chair height, increased time held in balance stances and advancement to more difficult static balance tasks, reduced support or increased height of stepping block. A booklet was provided containing the prescribed strength and balance exercises. Modifications to the format included large print and colour contrasted fonts to enhance appearance and highlight important information, colour photographs of correct technique and simplified written instructions. Exercises were always completed in the same place within participants’ homes and details, for example of where to stand or hold on, was recorded on each exercise sheet. |
| Type of intervention | Multifactorial; strength and balance exercises, home hazard reduction. |
| Duration of intervention | 12 weeks. |
| Frequency, timing, and duration of intervention contacts/visits | Six occupational therapy visits, five physiotherapy visits, three phone calls, baseline and post-intervention measurements. Occupational therapy visits on week one, three, five, seven and twelve. Physiotherapy visits on week two, four, six and eight. Phone calls on week nine, ten and eleven. The first two weeks had two visits/week while subsequent weeks had one.  The intervention group received a mean number of ten home visits (range 6–12) of 57.5 minutes duration and 3.5 telephone calls of 12.9 minutes duration, equating to 10.3 hours of direct contact per participant. Two people (18%) had fewer than ten visits: one person had only six visits as she declined the home safety intervention and the other was unwell after the seventh visit and discontinued intervention. |
| Description of control | Received usual care. They were encouraged to report any falls to their general practitioner and did not receive any further contact from the investigators except for collection of falls data and follow up assessment. Both intervention and control groups received health promotion brochures on fall prevention and home safety. |
| Follow-up beyond intervention length | N/A |
| Serious adverse events | No serious adverse events related to the intervention were reported during the study period. Minor complaints relating to stiffness, dizziness and mild joint pain (n=4; 36%) were reported by participants intermittently and exercises were adjusted accordingly. |
| Funding Source | New investigator grant from Alzheimer’s Association, USA and an Alzheimer’s Australia Research, Dementia Research Grant for new researchers. |

| **Feasibility, safety and preliminary evidence of the effectiveness of a home-based exercise programme for older people with Alzheimer’s disease: a pilot randomized controlled trial. Suttanon** | |
| --- | --- |
| Study (Year Published) | 2013 |
| Country | Australia |
| Objective/purpose | Investigated the feasibility and safety of a home-based exercise programme that focused on balance, strengthening and walking exercises, and to provide preliminary evidence of the effect of the programme on falls and physical performance in community-dwelling older people with mild to moderate Alzheimer’s disease. |
| Study design | Randomized controlled trial. |
| Recruitment setting and/or recruitment methods | Diagnosis, attended clinic, received support. Participants with Alzheimer’s disease were recruited through Memory Clinics at two large metropolitan hospitals, community groups providing support for people with Alzheimer’s disease and through public notices in newspapers. |
| Study setting and number of sites | Home-based. Number of sites N/A. |
| Level of analysis | Individual |
| Target population | Older individuals with Alzheimer’s disease. |
| Inclusion Criteria and Exclusion Criteria | Inclusion: diagnosis of Alzheimer’s disease confirmed from specialist or Memory Clinic assessment, if the Alzheimer’s disease symptoms were of mild to moderate severity (Mini Mental State Exam score ≥10) and if they could walk outdoors with no more support than a single-point stick. Participants were also living in the community and had no other serious orthopaedic condition (e.g. recent lower limb surgery, severe lower limb arthritis) or major neurological disorder (e.g. stroke, Parkinson’s disease) that could potentially restrict functional mobility.  Exclusion: N/R |
| Cognitive impairment tool | Diagnosis; Mini Mental State Exam score ≥ 10. |
| Baseline cognitive impairment | Overall score (SD): N/R  Intervention score (SD): 20.89 (4.74)  Control score (SD): 21.67 (4.43) |
| Total sample n (number eligible) | 40 (Five of 52 ineligible). |
| Intervention n (number invited) | 19 |
| Control n (number invited) | 21 |
| Participation rate | 40/52 = 76.9% |
| Attrition/Loss to follow-up: I n (%); C n (%) | Attrition rate of 27.5% (11/40).  I: 8 (42); C: 3 (14) |
| Age | Mean age overall (SD): 81.90 (5.72)  Mean age intervention (SD): 83.42 (5.10)  Mean age control (SD): 80.52 (6.01) |
| Gender: overall n (%); I n (%); C n (%) | Overall: Female: 25 (63); Male: 15 (37)  Female: I: 13 (68); C: 12 (57)  Male: I: 6 (32); 9 (43) |
| Race/Ethnicity | N/R |
| SES status*(reported by income or education level ONLY)* | N/R |
| Co-morbidities/chronic conditions | Number of medical conditions, median (range):  I: 3 (6); C: 3 (5) |
| Description of intervention | Provided with a six-month individualized home-based exercise programme supervised by a physiotherapist. The programme included standing balance and strengthening exercises, a graduated walking programme and was based on the Otago Program. The physiotherapist made six home visits which provided increased support throughout the six-month duration, and maximized the participants’ and caregivers’ understanding of the exercises and of safety issues, particularly during the early phase of the programme. Each participant also received an exercise booklet with illustrations and instructions, and was encouraged to complete the exercises five times/week. At the first visit, the physiotherapist selected and modified exercises from the Otago Program to address the individual’s balance and mobility problems as identified in the baseline balance and mobility assessment. At each subsequent home visit, the physiotherapist monitored and modified the exercise programme as required and answered any questions. Caregivers were also instructed regarding the exercise programme and asked to encourage regular (five days/week) and correct performance of the exercises. Follow-up phone calls by the physiotherapist in between visits were also provided to offer reassurance, to enquire if there were any negative effects from the exercises (such as falling or other physical injuries) and to answer questions about the exercises (five phone calls over the six-month period). The participants and their caregivers were also provided with the physiotherapist’s contact telephone details and were told that they should contact the physiotherapist if they had any questions or concerns about the programme. |
| Type of intervention | Exercise; balance and strength exercises, walking program. |
| Duration of intervention | Six months. |
| Frequency, timing, and duration of intervention contacts/visits | Six physiotherapy visits, five phone calls, two measurement visits. Timing and duration of visits were N/R. |
| Description of control | Provided with the same number of home visits and phone calls as the exercise programme. It consisted of education and information sessions on the topic of dementia and ageing that were not anticipated to influence the primary physical performance outcomes of the study. The education and information programme was delivered by an occupational therapist and was based in part on a previously reported home-based education and support programme for people with mild to moderate dementia and their caregivers.  The first visit was designed to educate and clarify any possible misunderstanding about Alzheimer’s disease. During the second visit, the therapist talked about potentially useful aged care services that were available in the participant’s municipality. At the third visit, the therapist discussed the participant’s previous and current functioning and their participation in activities of daily living. Practical advice given included recommendations of memory strategies and breaking activities into tasks to identify which ones the participant could achieve, then delegating remaining tasks to the caregivers or others. During the fourth visit, the therapist discussed on average two–three issues of concern to the participant/caregiver identified in the preceding sessions (e.g. behavioural and psychological symptoms of dementia, anxiety and medication) and supplemented this with sourced printed material. At the fifth session, the therapist discussed and provided printed information on topics about enduring powers of attorney, advanced care directives, wills and residential care options. At the final visit, the therapist reviewed significant issues of interest to the participant and their caregiver that were identified during the previous visits. |
| Follow-up beyond intervention length | N/A |
| Serious adverse events | There were no falls or other serious adverse events associated with performing the exercise programme. Several participants, however, reported pain or discomfort when a new exercise was introduced. However, those symptoms either eased with continuing the exercises or were resolved by slight modification of the exercise by the physiotherapist. |
| Funding Source | National Ageing Research Institute. |

| **Effects of physical activity on cognitive functions, balance and risk of falls in elderly patients with Alzheimer’s dementia. Hernandez.** | |
| --- | --- |
| Study (Year Published) | 2010 |
| Country | Brazil |
| Objective/purpose | Analyzed the effect of a regular, systematic and supervised program of physical activity on cognitive functions, balance and risk of falls in patients with Alzheimer’s dementia. |
| Study design | Clinical controlled trial. |
| Recruitment setting and/or recruitment methods | All participants had been diagnosed with Alzheimer’s dementia according to the criteria of the Diagnostic and Statistical Manual of Mental Disorders (DSMIV). The selected participants volunteered for the study or were referred by physicians and caregivers. |
| Study setting and number of sites | N/R |
| Level of analysis | Individual |
| Target population | Older individuals with Alzheimer’s dementia. |
| Inclusion Criteria and Exclusion Criteria | Inclusion: N/R  Exclusion: N/R |
| Cognitive impairment tool | Clinical Dementia Rating scale score. Diagnosis; Diagnostic and Statistical Manual of Mental Disorders criteria. Mini Mental State Exam. |
| Baseline cognitive impairment | Overall score (SD): N/R  Intervention score (SD): 16.4 (6.7)  Control score (SD): 14.2 (5.1) |
| Total sample n (number eligible) | 20 (N/R) |
| Intervention n (number invited) | 9 |
| Control n (number invited) | 7 |
| Participation rate | NR |
| Attrition/Loss to follow-up: I n (%); C n (%) | Attrition rate of 20%.  Initially 20 elderly patients took part in this study. However, there was sample loss of four elderly patients due to health problems, followed by hospitalization. Thus, the study was completed with 16 elderly patients with Alzheimer’s Dementia (I: n=9; C: n=7) |
| Age | Mean age overall (SD): 78.5 (6.8)  Mean age intervention (SD): 77.7 (7.6)  Mean age control (SD): 84.0 (6.1) |
| Gender: overall n (%); I n (%); C n (%) | Overall: N/R  Female: N/R  Male: N/R |
| Race/Ethnicity | N/R |
| SES status*(reported by income or education level ONLY)* | Schooling, years (SD):  I: 5.6 (3.3); C: 4.5 (3.0) |
| Co-morbidities/chronic conditions | N/R |
| Description of intervention | The systematic and supervised physical activity program consisted of 60-minute sessions performed three times/week on non-consecutive days for six months. The sessions were performed in groups with the help of trainees. This program was prescribed according to the functional and cognitive capacity of the elderly participants, aimed at maintaining independence and reducing the risk of falls. The exercises were structured to promote the motor and cognitive stimulation of the participants, simultaneously or separately. For motor development, stretching activities, weight training, circuits, pre-sport games, dance sequences, recreational activities and relaxation were prescribed. Auxiliary apparatuses were used (e.g. weights, ankle weights, sticks, medicine ball, thera-band and gymnastics ball) to develop components of the functional capacity of coordination, agility, balance, flexibility, strength and aerobic capacity. During the sessions, the participants’ heart rate was measured with a Polar A4 heart rate monitor, therefore the training was adapted so as to require an effort equivalent to 60-80% of the maximum heart rate. |
| Type of intervention | Exercise; stretching, weight training, circuits, dance, recreational activities, relaxation. |
| Duration of intervention | Six months. |
| Frequency, timing, and duration of intervention contacts/visits | Three sessions/week for six months (72-75 total sessions), pre and post measurement visits. The duration of each session was 60 minutes. The duration of measurements was N/R. |
| Description of control | Maintained their pharmacological assistance and medical routine without changes. Participants did not follow the exercise protocol. |
| Follow-up beyond intervention length | N/A |
| Serious adverse events | N/R |
| Funding Source | CAPES, LAFE and LEPLO (Unesp - IB - Rio Claro); FINEP; FNS-MS; FUNDUNESP; PROEX-UNESP. |
